# Engineering Motile Coacervate Droplets via Nanomotor Stabilization

**DOI:** 10.1021/jacs.5c09366

**Published:** 2025-08-20

**Authors:** Siwen Sun, Jianhong Wang, Yudong Li, Alexander B. Cook, Bingbing Sun, Sebastian Novosedlik, Lars J. M. M. Paffen, Sander G. A. M. Huisman, Lou M. V. Raeven, Alexander D. Fusi, Yunqi Guo, Loai K. E. A. Abdelmohsen, Shukun Li, Tania Patiño Padial, Jan C. M. van Hest

**Affiliations:** † Bio-Organic Chemistry, Departments of Biomedical Engineering and Chemical Engineering & Chemistry, Institute for Complex Molecular Systems, 3169 Eindhoven University of Technology, 5600 MB Eindhoven, The Netherlands; § State Key Laboratory for Modification of Chemical Fibers and Polymer Materials, Shanghai Engineering Research Center of Nano-Biomaterials and Regenerative Medicine, College of Biological Science and Medical Engineering, 12475 Donghua University, Shanghai 201620, PR China

## Abstract

Coacervate-based artificial cells have gained significant attraction in synthetic biology for their ability to mimic life-like functions such as compartmentalization, selective molecular uptake, and the hosting of biochemical reactions. However, the incorporation of motility, a key feature of natural cells, remains underexplored. This is mainly caused by the dynamic character of coacervates, which hampers their stability and limits control over functional motile components within the structure. In this contribution, we have been able to address this gap by physically anchoring gold nanoparticles (AuNPs)-coated nanomotors at the coacervate interface in combination with a terpolymer membrane. The positively charged coacervates promoted the assembly of negatively charged nanomotors on their surfaces via electrostatic interactions. By costabilizing the coacervates with a terpolymer membrane, patches of nanomotors were firmly immobilized on the coacervates’ surface and the stability of coacervates was preserved during motion performance. The distribution of nanomotors shifted from spotted distribution, patchy distribution, to almost full coverage upon increasing nanomotors concentration. Optimal motile behavior was found when achieving a patchy coverage of nanomotors at the interface, which enabled the system to become a light-driven micromotor platform through the surface plasmon thermal effect of the AuNPs. Remarkably, the motion dynamics of these coacervate droplets could be modulated by tuning nanomotors’ density on the surface, coacervates’ size, and laser light intensity. This study provides a first example of a coacervate system, which is stabilized by a combination of nanoparticles and a terpolymer membrane, of which their motility is effectively transferred to the artificial cell structure.

## Graphical abstract

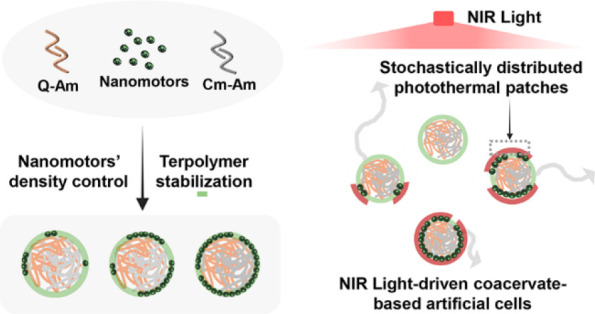

## Introduction

Artificial cell research is an emerging field that focuses on building biomimetic systems from the bottom up, using a combination of synthetic and natural components to replicate the structure and function of living cells.^1–3^ Besides the fact that this approach provides more fundamental insight into how living cells operate, artificial cell platforms can also be applied in different fields, including medicine, biotechnology, and environmental engineering.^1,4,5^

Complex coacervates have been intensively studied as an artificial cell platform as they mimic well the crowded intracellular environment of the cytoplasm.^6^ Coacervates are typically formed when oppositely charged polyelectrolytes condense into a polymer-rich phase.^6–8^ Traditionally, these coacervate microdroplets are created without a protective boundary, which makes them, however, susceptible for coalescence and surface wetting. To improve stability and enhance cell-mimetic characteristics, different membranization approaches have been developed.^9–13^ In our group, we created terpolymer-stabilized amylose-based coacervates.^13^ This system offers improved mechanical stability while allowing the exchange of biomolecules. To further increase the structural mimicry of this platform, we have also been able to include polymersomes as artificial organelles within the coacervate phase and have endowed them with an artificial cytoskeleton.^13–15^ Coacervate membranization is not limited to amphiphilic molecules. An alternative method for stabilization is the use of nanoparticles, which adhere at the interface.^11,16^ Although different types of particles have been employed, nanoparticles with motile features have not yet been investigated as interfacial components of coacervates.

Despite remarkable progress in the development of coacervate-based artificial cells—particularly in emulating key biological attributes such as hierarchical compartmentalization,^17,18^ intercellular communication,^19,20^ and rudimentary metabolic functions,^21^ the incorporation of motility, a defining characteristic of living systems, remains relatively underexplored. While notable advances have been made, such as motile artificial cells constructed from enzyme-conjugated terpolymer membrane-stabilized droplets and electrically driven droplets exhibiting high polarizability,^22–24^ a fundamental challenge persists: the precise integration and spatial control of motile elements within the coacervate matrix without compromising the structural integrity and dynamic stability of the system.

In this study, we aimed at addressing this important gap by embedding light-driven nanomotors into the terpolymer membrane of our coacervate platform to introduce both stability and motility to these artificial cells (Figure 1). The nanomotors were based on bowl-shaped polymer vesicles, or stomatocytes, which were coated on the outer surface with small gold nanoparticles (AuNPs). In earlier research, we demonstrated that the asymmetric shape of these stomatocytes enabled a temperature gradient to be formed when AuNPs were irradiated with NIR light.^25^ Here, we showed that the nanomotors were embedded in the terpolymer membrane in a stochastic way, and their density on the coacervate surface could be controlled by increasing the nanomotor:coacervate ratio. Furthermore, in contrast to the terpolymer, the nanomotors were immobilized on the coacervate surface. Coacervates were also stabilized by a full coverage of the surface with nanomotors in the absence of terpolymer. For optimal motile behavior, however, patchy coverage was required, which was obtained when the nanomotors were combined with the terpolymer membrane.

**Figure 1:**
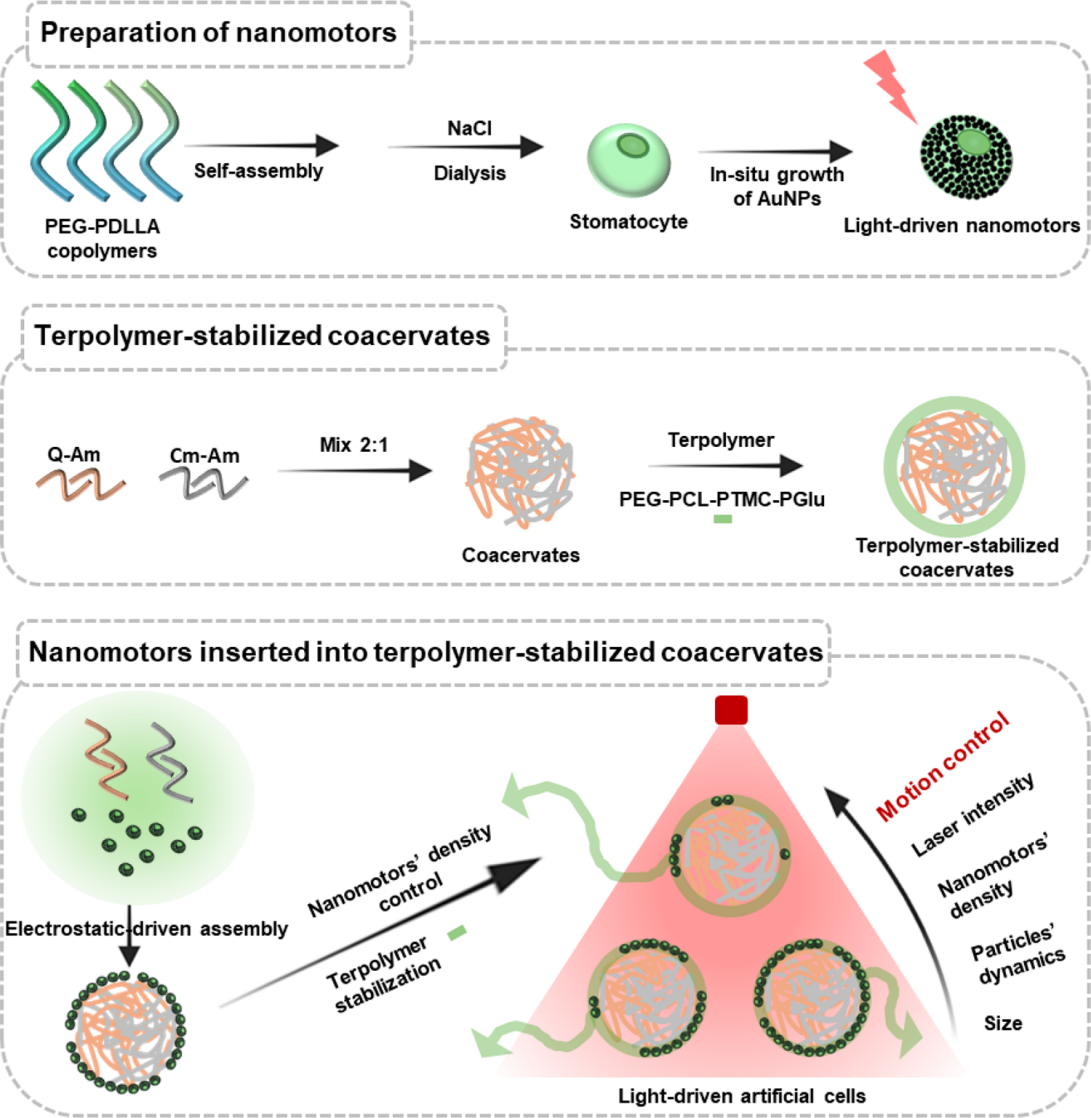
Schematic illustration of the preparation of nanomotors inserted into terpolymer-stabilized coacervates as a light-driven artificial cell platform.

## Results and Discussion

### Formation of Hybrid Coacervates with Nanomotors at the Interface

For the preparation of the nanomotors, a procedure was followed previously reported by our group (Figure S1).^25,26^ Briefly, PEG-PDLLA block copolymers (PEG_22_-PDLLA_95_ and PEG_44_-PDLLA_95_) were synthesized and subsequently assembled into polymersomes. After dialysis-induced shape changes into stomatocytes, the surface was efficiently decorated with gold nanoparticles (AuNPs) to yield the desired nanomotors. The charge, size, and morphology of the stomatocytes and nanomotors were characterized using dynamic light scattering (DLS), scanning electron microscopy (SEM), and cryogenic transmission electron microscopy (cryo-TEM) (Figure 2a and Figures S2 and S3). Cryo-TEM analysis demonstrated the effective decoration of AuNPs on the stomatocyte surface. DLS results indicated that both size and surface negative charge of the stomatocytes increased after AuNPs deposition, from 308 to 340 nm and from −20 to −34 mV, respectively. To visualize the nanomotors using confocal laser scanning microscopy (CLSM), a Cy5.5 dye was encapsulated within the nanomotors, which were observed to be well dispersed in aqueous solution (Figure 2b).

**Figure 2:**
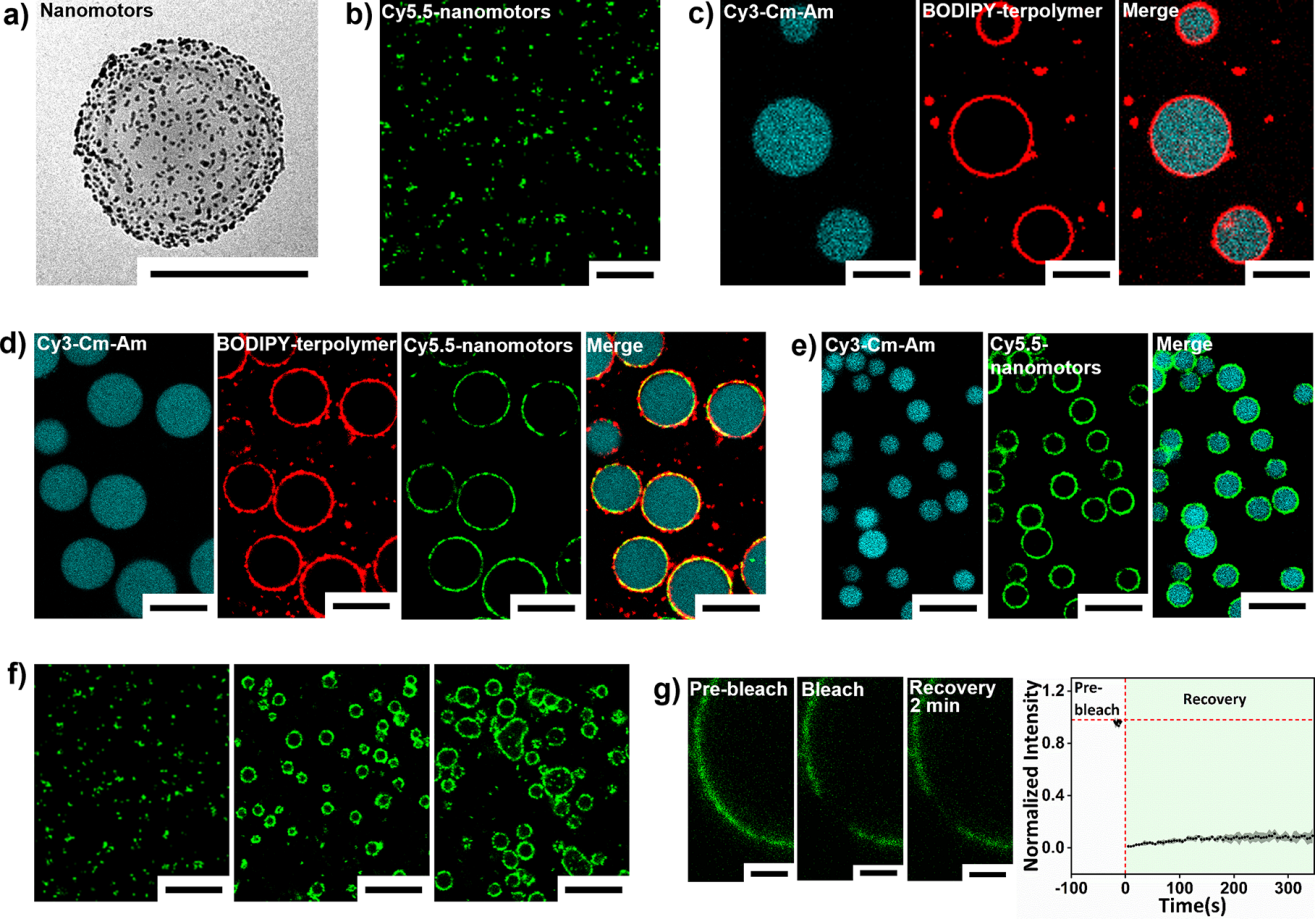
(a) Cryo-TEM analysis of AuNPs-decorated stomatocyte nanomotors. (b) CLSM images of well-dispersed nanomotors loaded with the dye Cy5.5. (c) CLSM images of terpolymer-stabilized coacervates. (d) CLSM images of terpolymer-stabilized coacervates with nanomotors at the interface (3 μL of 50 mg/mL terpolymer solution, 30 μL of 3.33 mg/mL nanomotor solution). (e) CLSM images of nanomotor-stabilized coacervates without terpolymer (50 μL of 3.33 mg/mL nanomotor solution). (f) CLSM images of Cy5.5-labeled stomatocyte-stabilized coacervates (left) and LBL-modified stomatocyte-stabilized coacervates (middle and right); the concentrations of modified solution (PAH/PSS) were 2 mg/mL (middle) and 10 mg/mL (right). A 50 μL 3.33 mg/mL stomatocyte solution (left) and 50 μL of 3.33 mg/mL LBL-modified stomatocyte solution (middle and right) were added. No terpolymer was present. (g) FRAP measurements of terpolymer-stabilized coacervates with fluorescently labeled nanomotors at the interface (3 μL of 50 mg/mL terpolymer solution, 30 μL of 3.33 mg/mL Cy5.5-labeled nanomotor solution). FRAP measurements were conducted on three different coacervate droplets separately. Data were shown as mean ± standard deviation. Green: nanomotors loaded with Cy5.5, cyan: Cy3 conjugated *Cm*-Am, red: BODIPY stained the terpolymer. Scale bars represent 250 nm (a), 10 μm (b–f), and 3 μm (g). Images were adjusted for brightness and contrast solely for the visualization of each component. No quantitative analysis was performed on these processed images (b–f). FRAP data analysis was performed using raw unprocessed images (g).

Coacervates were then prepared using the method previously reported by our group.^7,27^ Coacervation was initiated by mixing positively charged amylose (modified with a quaternary amine, Q-Am) with negatively charged amylose (modified with a carboxymethyl group, *Cm*-Am) in phosphate-buffered saline (PBS).^7^ After shaking for 4 min, the terpolymer poly­(ethylene glycol)-*b*-poly­(ε-caprolactone)-*g*-poly­(trimethylene carbonate)-*b*-poly­(glutamic acid) (PEG_44_-*b*-PCL_50_-*g*-PTMC_50_-*b*-PGA_8_) was introduced, which assembled on the surface of the nascent coacervate droplets, stabilizing them against coalescence (Figure 2c). To obtain coacervates stabilized by terpolymer while nanomotors were introduced, the nanomotors were first added to the *Cm*-Am solution, after which this mixture was combined with Q-Am to initiate coacervation. The terpolymer was then introduced to halt droplet growth. For visualization, a Cy3 dye was conjugated to *Cm*-Am, and BODIPY was used to stain the terpolymer membrane, enabling identification of the components of the hybrid coacervates under CLSM. CLSM images revealed that the core of the hybrid coacervates consisted of the two oppositely charged amylose components, while the nanomotors and terpolymer remained at the coacervate interface after self-assembly (Figure 2d and Figure S4). To determine if the remarkable preference of the nanomotors for the interface was a result of the coacervate preparation method, we also directly mixed the three components (C*m*-Am, Q-Am, and nanomotors) in a PBS buffer solution at a volume ratio of 33:66:50 (Figure 2e). CLSM images demonstrated that the core of the droplets again consisted of the two oppositely charged amylose components, while the nanomotors densely arranged to form the membrane structure of the droplets (Figure S5). In addition to Cy5.5, other fluorescent dyes (FITC and DiD) were also successfully encapsulated in the nanomotors to confirm the interfacial assembly of nanomotors around the coacervate droplets (Figure S6). These results proved that *Cm*-Am and Q-Am formed the coacervate core via liquid–liquid phase separation and the nanomotors spontaneously assembled onto the amylose-based coacervate interface independent of the sequence in which the components were added. Compared to other reports on particle-stabilized coacervates, such as BSA-stabilized MnO_2_ nanoparticles,^28^ Au/PEG nanoparticles,^16^ and bare liposome^29^-stabilized coacervates, this study represents the first assembly of larger-sized motile particles (larger than 340 nm) at the interface of coacervate droplets.

Previous research demonstrated that coacervates possess a strong encapsulation ability for various components, such as fluorescent dyes,^7^ proteins,^7,13^ and vesicles (e.g., polymersomes^14^ and liposomes^30^) through electrostatic absorption. An explanation for the preferential location of the nanomotors at the interface was found in their large size and, in particular, the negative zeta potential, which reduces their ability to effectively participate in coacervate formation via liquid–liquid phase separation. To verify this hypothesis, we investigated the effect of the surface charge on the interfacial assembly. For this purpose we attempted to stabilize coacervates with bare PEG-PDLLA stomatocytes, which were less negatively charged than the AuNPs-coated variants (Figure S3). The bare particles failed to assemble at the coacervate interface and remained well dispersed in the solution (Figure 2f). To increase the PEG-PDLLA stomatocytes’ surface charge, they were coated using a Layer-by-Layer (LBL) assembly approach with, respectively, cationic polyelectrolyte (PAH) and anionic polyelectrolyte (PSS). First, PAH solution was added to the stomatocytes templates. After washing, PSS was applied as the second modification layer. This treatment resulted in stomatocytes with a significantly increased negative zeta potential of −32.7 mV (Figure S7).^31–33^ These PAH-PSS LBL-modified stomatocytes were also observed to achieve interfacial assembly around the coacervate droplets, confirming the important role of electrostatic interactions during interfacial adsorption (Figure 2f). When the negative charge of the modified stomatocytes was further increased by increasing the concentration of PSS and PAH solution (from 2 to 10 mg/mL) via the LBL approach to reach −41 mV, some of the coacervate droplets deformed, possibly due to the adsorption of modified stomatocytes into the interior of the coacervates, disrupting the system’s interfacial balance. Hence, the increased negative charge played an important role in the remarkable preference of the nanomotors for the coacervate interface, which was attributed to the electrostatic interaction between negatively charged nanomotors and positively charged Q-Am.

Another strategy for modulating electrostatic interactions involves adjusting the ratio of Q-Am to *Cm*-Am. Specifically, we investigated whether increasing the positive charge of the coacervates—by raising the volume ratio of Q-Am to *Cm*-Am—could enhance the interfacial adsorption of bare PEG-PDLLA stomatocytes. To this end, we mixed bare stomatocytes with Q-Am and *Cm*-Am at varying volume ratios (Q-Am:*Cm*-Am= 3:1, 5:1, 7:1, and 9:1). However, stable coacervate droplets were not observed at any of these ratios, even at the highest Q-Am:Cm-Am ratio of 9:1 (Figure S8). A plausible explanation is that when the overall positive charge of the coacervates remains insufficient (i.e., the Q-Am content is still below the required level), the electrostatic attraction is not strong enough to drive the effective adsorption of bare stomatocytes at the coacervate interface. Conversely, even when the positive charge is high enough to promote adsorption, insufficient electrostatic repulsion between droplets stabilized by bare stomatocytes may result in instability of coacervates—manifested as coalescence or even droplet rupture.^22,34^ Therefore, tuning the Q-Am/*Cm*-Am ratio alone was not sufficient to achieve stable stomatocyte-stabilized coacervate in our system.

The membrane of the terpolymer-stabilized coacervates was shown to be highly fluidic, as a result of the polymer’s low glass transition temperature (*T*_g_).^7,35^ To investigate if also the nanomotors were dynamic at the coacervate interface, we performed fluorescence recovery after photobleaching (FRAP) experiments on the fluorescently labeled nanoparticles when they were coassembled with the terpolymer at the interface. No significant fluorescence recovery was observed in the bleached area, suggesting that the nanomotors were firmly anchored on the surface of the coacervates and unaffected by the fluidity of the terpolymer, likely due to the nanomotors’ large size and strong electrostatic anchoring (Figure 2g).

### Variation of Nanomotor Density at the Interface of Coacervates

As shown in Figure 2e, coacervates were effectively stabilized by the nanomotors even in the absence of terpolymer. A more detailed 3D confocal z-stack image (Figure S9) demonstrated that the stabilization layer was discontinuous with small holes and defects. Next, we determined this stabilization effect as a function of nanomotor concentration. When a low amount of nanomotors was introduced (3.33 mg/mL, <30 μL), the coacervates tended to collapse on the glass slide due to incomplete surface coverage (Figures S10–S12). Only when >50 μL of nanomotors was added, stable coacervates were obtained. However, we hypothesized that this full coverage did not facilitate effective motion of coacervates, as the forces exerted by the nanomotors should counterbalance each other. Lower nanomotor coverage at the interface was therefore thought to be more beneficial for decreasing the likelihood of counteracting forces. To achieve this, we further investigated the mixed system in which both terpolymer and stochastically distributed nanomotors were present at the coacervate interface. For the visualization with CLSM, the nanomotors were loaded with Cy5.5 and coacervates with FITC; the terpolymer was stained with Nile red.

As shown in Figure 3a–d, in the presence of terpolymer, coacervates were stable under all measured nanomotor concentrations. We observed that the nanomotors were embedded within the terpolymer membrane, and the embedding within the membrane also showed stochasticity (Figure 3e,g,i). Also consistent with the observations of Figure 3b–d, more nanomotors were embedded in the terpolymer membrane as the concentration of nanomotors increased. This spatial embedding was further illustrated by the 3D confocal z-stack images (Figure 3f,h,j). When introducing a limited amount of nanomotors (5 μL, 3.33 mg/mL), nanomotors exhibited a spotted distribution at the coacervate interface, which changed into patchy coverage at a higher concentration of nanomotors (15 μL, 3.33 mg/mL). When a sufficient amount of nanomotors (30 μL, 3.33 mg/mL) was absorbed at the interface, nanomotors dominated the hybrid membrane, and the terpolymer just filled the small interfacial voids that were not covered by the nanomotors. In addition, fluorescence intensity line profiles along the perimeter of coacervate droplets were also used to assess the distribution of the nanomotor patches. Consistent with the confocal microscopy images, the line profile results confirmed that the anisotropy in nanomotor distribution on the coacervate interface decreased with increasing nanomotor density (from 5-Coas to 30-Coas). This process is always controlled by the stochastic interfacial adsorption of nanomotors (Figure S13). The patch-like hybrid membrane effectively stabilized the coacervates for over 1 day, maintaining their size and morphology without any noticeable changes (Figure S14).

**Figure 3:**
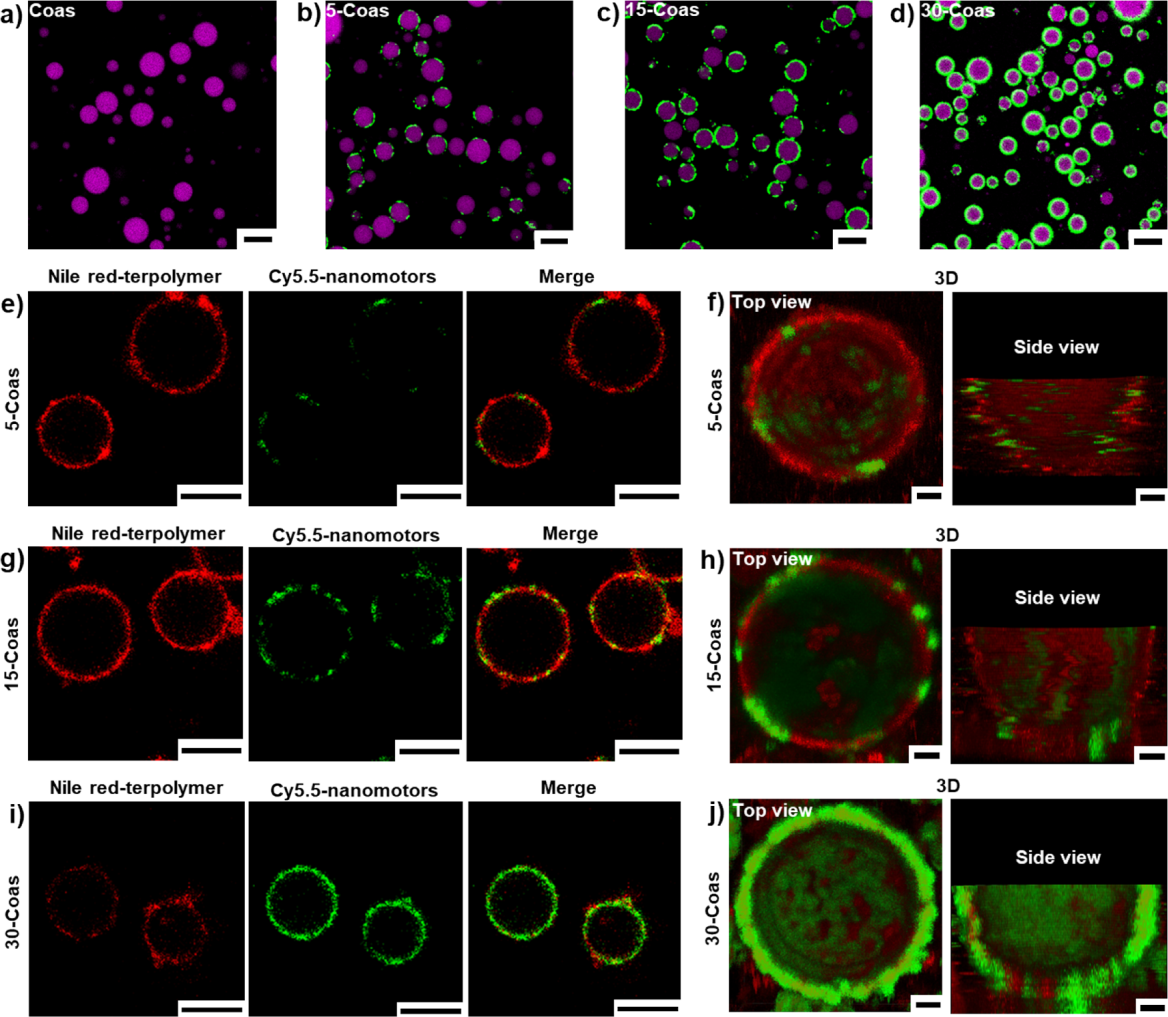
CLSM images of terpolymer-stabilized coacervates loaded with fluorescent dye FITC, with or without nanomotors at the interface. (a) 0 μL of 3.33 mg/mL nanomotor (Coas), (b, e) 5 μL of 3.33 mg/mL nanomotor (5-Coas), (c, g) 15 μL of 3.33 mg/mL nanomotor (15-Coas), and (d, i) 30 μL of 3.33 mg/mL nanomotor (30-Coas). 3D confocal z-stack micrographs of (f) 5-Coas, (h) 15-Coas, and (j) 30-Coas. Top view (left) and side view (right). Amylose-based coacervates are shown in magenta, nanomotors are loaded with Cy5.5 (green), and the membrane is stained with Nile red (red). Scale bars represent 10 μm (a–d), 5 μm (e, g, and i), and 1 μm in 3D confocal z-stack micrographs. Panels (a)–(d) were processed using identical brightness and contrast settings to ensure comparability within this group. Similarly, panels (e), (g), and (i) were adjusted with another set of consistent brightness and contrast parameters, and panels (f), (h), and (j) were processed by using a third consistent set. All images were intended solely for visualization purposes and were not used for quantitative fluorescence analysis.

### Permeability of the Terpolymer Membrane with Inserted Nanomotors

Next, we assessed how variations in the interfacial components influence the permeability of coacervate droplet membranes as increasing the number of nanomotors incorporated into the hybrid membrane could potentially alter molecular transport. Gaining such insights is important for understanding the broader functional capabilities of coacervates, particularly in applications involving chemical communication, compartmentalized reactions, or targeted cargo exchange.^11^ We studied this by using FITC-labeled dextran polymers with varying molecular weights. The degree of coacervate permeability was determined by quantifying their encapsulation efficiency by confocal microscopy. We noted a size-dependent effect on the sequestration of molecular cargos into coacervates for both terpolymer-stabilized coacervates and terpolymer/nanomotor-stabilized coacervates, which was consistent with previous reports.^7,35^ The larger the size of the molecular cargo, the more difficult it was to achieve transmembrane uptake (Figure S15a–d). The 4 kDa dextran was easily taken up by coacervates, while the medium-sized 40 and larger 500 kDa dextran were significantly excluded. Interestingly, we observed that the density of nanomotors on the coacervate surface also played a role on permeability. For coacervates with lower amounts of nanomotors in the membrane (5- and 15-Coas), their spotted and patchy distribution did not significantly affect the coacervate’s permeability, as dextran was still able to penetrate the terpolymer membrane in regions without nanomotors. However, coacervate interface displayed an almost full coverage by nanomotors (30-Coas), the permeability for dextran was significantly lower than that of the terpolymer membrane after 10 min incubation, and this was consistent for all types of dextran cargoes (Figure 15e). To obtain deeper insights into the permeability dynamics of fully nanomotor-coated coacervates, we monitored dextran uptake overtime. After 24 h, no significant variance in the encapsulation efficiency for 4 kDa dextran was observed, which confirmed the rapid uptake characteristics for this small-sized dextran (Figure S15f).^35^ For medium-sized 40 kDa dextran, we observed improved sequestration efficiency comparing the 10 min and 24 h time points, indicative that bigger dextran had slower uptake kinetics (Figure S15g). The insignificant variance for the larger 500 kDa dextran over a 24 h period showed its strong exclusion effect (Figure S15h). These results demonstrated that the insertion of nanomotors into the terpolymer membrane significantly reduced the permeability to dextran when a sufficient number of nanomotors were embedded within the membrane. Although the insertion of additional nanomotors may introduce structural defects, this effect is effectively mitigated by the subsequent addition of terpolymer molecules, which help to fill the voids and stabilize the membrane. Furthermore, the dense arrangement of nanomotors at the coacervate interface may physically hinder the diffusion of guest solutes, contributing to the observed decrease in the permeability.

### Motion Analysis of Hybrid Coacervate-Based Artificial Cells

After having established the possibility to construct coacervates with controllable nanomotor coverage, we investigated their motion dynamics in the presence of light irradiation. We first examined the nanomotor’s photothermal properties by irradiating the samples with a 660 nm laser (1.5 W, 10 min). The temperature of the samples with nanomotors inserted into the terpolymer membrane significantly increased, compared with bare coacervates, and showed a dependence on the nanomotor quantity. For 30-Coas, the maximum temperature increase reached up to 24.4 °C within 10 min (Figure 4a). Additionally, the temperature increase of the hybrid coacervates was also proportional to the light intensity, with higher light intensity resulting in a larger temperature increase (Figure 4b).

**Figure 4:**
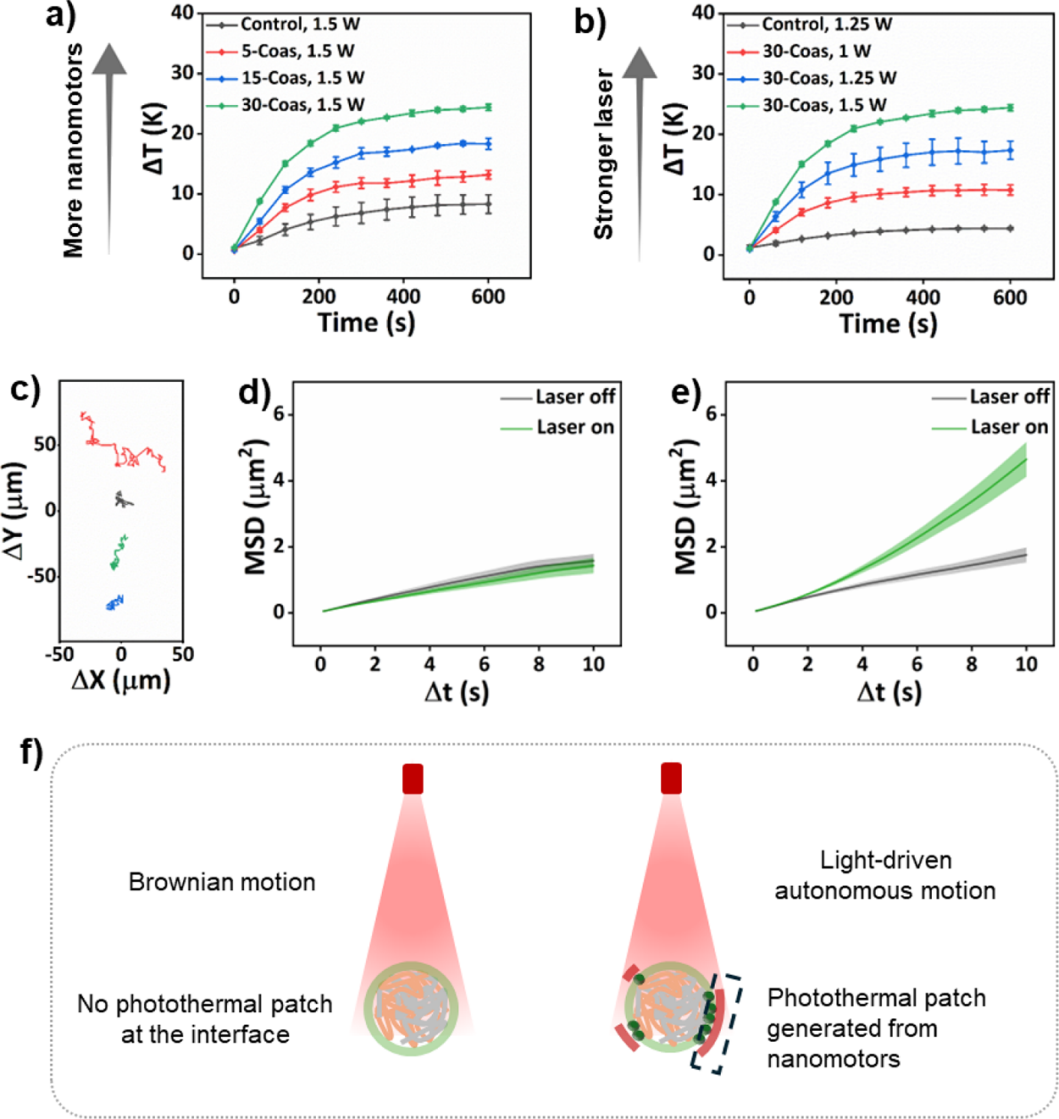
Light-driven motion behavior observed for coacervates with nanomotors inserted into the terpolymer membrane. (a) Temperature changes of hybrid coacervates with different nanomotor concentrations upon laser irradiation (660 nm, 1.5 W, 10 min). (b) Temperature changes of 30-Coas upon TP laser irradiation with different laser intensity (660 nm, 10 min); data represent mean ± SD for three independent samples. Error bars represent the standard deviation. (c) Representative trajectories of coacervate droplets: 35 s without laser irradiation and more than 2 min with 800 nm medium-intensity (30%) TP laser irradiation (black: a 5-Coas coacervate without laser irradiation, red: a 5-Coas coacervate under laser irradiation, blue: a bare coacervate without laser irradiation, and green: a bare coacervate under laser irradiation). MSD curves of bare coacervates (d) and 5-Coas (e) without and with medium-intensity (30%) TP laser irradiation. (f) Schematic illustration of the self-propulsion of coacervates in solution. Active patches (AuNPs-coated nanomotors) embedded stochastically inside the coacervate membrane generated asymmetric plasmonic heating under laser activation, which eventually promoted the movement of the entire coacervate hybrid coacervate system. More than 16 coacervates were analyzed per condition. MSD data are presented as mean ± SEM. The size of all coacervates was about 3.6 μm.

Subsequently, the motility of the coacervates was recorded without and with laser irradiation using a confocal microscope equipped with a two-photon laser (TP-CLSM, λ_ex_ = 800 nm). Coacervates with a diameter of approximately 3.6 μm were selected for motility experiments (Figure S16). We first studied the *x*–*y* trajectories of coacervates coated with a low density of nanomotors in their membrane (5-Coas). These coacervates displayed a notable enhanced diffusion under TP laser irradiation compared to bare coacervates (Figure 4c and Movie S1). Using a custom Python script, we calculated the mean square displacement (MSD) from the obtained trajectories.^23^ For bare coacervates, typical Brownian motion with linear MSD fitting profiles was observed without and with laser irradiation, with MSD values remaining similar at a 10 s interval (Figure 4d). For 5-Coas, a clear parabolic MSD profile was observed (Figure 4e), indicating a ballistic motion. The random patchy distribution of AuNPs-coated nanomotors firmly fixed on the coacervates’ surface resulted therefore in light-activated asymmetric plasmonic heating localized at the AuNPs surface, thereby promoting the movement of the entire hybrid coacervate system (Figure 4f). In addition, we examined whether the configuration of nanomotor patches changed during motion. To this end, we performed 3D confocal z-stack imaging before laser activation and again after a 2 min period of light-induced motion, and subsequently compared the spatial distribution of the nanomotor patches (Figure S17 and Movie S2). The results revealed no significant dynamic rearrangement over the course of motion. The positions of the nanomotor patches remained largely unchanged, indicating that their configuration is stable throughout the motility process. These observations support our proposed self-thermophoretic mechanism, in which a static asymmetric distribution of nanomotor patches leads to autonomous motion through asymmetric local heating.

Asymmetry in the motor structure, such as variations in shape or catalyst distribution, is considered a crucial prerequisite for enabling autonomous motion.^36–38^ As we discussed earlier, we were able to control the distribution of nanomotors at the interface of the coacervates, from a spotted distribution via patchy distribution to almost full coverage. Throughout this process, the stochastic nature of nanomotor adsorption at the coacervate interface inherently leads to anisotropic surface distributions. This is particularly expected given that perfect isotropy is statistically improbable due to the inherently stochastic nature of the adsorption process (more obvious for 5-Coas and 15-Coas), especially on a three-dimensional spherical surface such as that of a coacervate droplet. Consequently, a certain degree of anisotropy naturally emerges. Under light irradiation, this anisotropic nanomotor arrangement at the coacervate interface induces asymmetric plasmonic heating, which subsequently drives the motion of entire coacervate droplets. Motivated by this, we further investigated how variations in nanomotor distribution patterns—specifically, different degrees of anisotropy—affect the motility of coacervates (Figure 5a). Coacervates with a patchy nanomotor distribution (15-Coas and 5-Coas), the coacervates exhibited significantly higher motion capabilities compared to fully covered coacervates. This is evident by the significant increase in MSD profiles (Figure 5b,c, Figure S18, and Movies S1 and S3). Interestingly, coacervates with a lower amount of nanomotors (5-coas) displayed higher motion capabilities than coacervates with medium coverage (15-coas), which could be explained by the increase in anisotropy. As expected, the control group without nanomotors (Coas) showed the lowest MSD values. From the MSD curves, we extracted the speed and effective diffusion coefficient (Deff) (calculated from MSD curves’ quadratic fitting). We observed that coacervates with almost full nanomotor coverage (30-Coas) showed significantly lower values for both speed and diffusion when compared to patchy nanomotor-coated coacervates (Figure 5d,e). Although the heating capacity at the coacervate interface was increased with the increase in nanomotors, the homogeneous nature of the heat distribution around the coacervates reduced their motile behavior (Figure 5a and Figure S13).^23,39^ This was in line with our earlier observations on an enzyme-decorated motile artificial cell system.^39^ With the current system, the optimal motion behavior occurred with the lowest number of motors loaded onto the surface of the coacervates, which was different from that of the enzyme-driven coacervates, in which a medium enzyme density induced the optimal motion. This difference can be explained by the power of the individual motor units, which is much higher for the AuNPs-decorated stomatocytes than for the enzymes employed in previous systems. Therefore, we hypothesize that, in this case, 5-Coas displayed a higher anisotropic gradient in temperature, leading to stronger motion effects.

**Figure 5:**
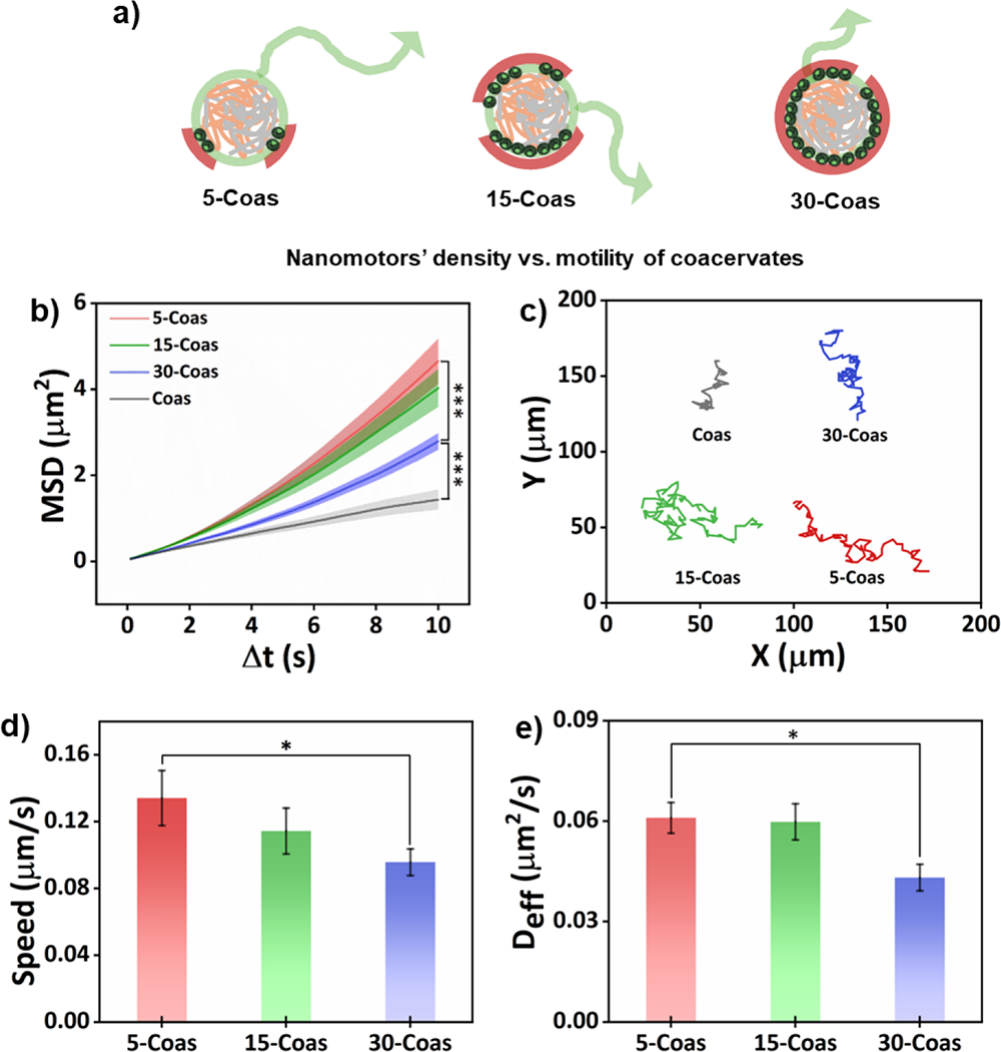
Investigation of coacervates’ motion dynamics depending on the nanomotor density at the interface. (a) Schematic illustration of stochastic asymmetry-induced motion of hybrid coacervates. (b) MSD curves and (c) corresponding trajectories of Coas, 5-Coas, 15-Coas, and 30-Coas with TP laser irradiation. (d) Corresponding velocity and (e) Deff of 5-Coas, 15-Coas, and 30-Coas with TP laser irradiation, which are derived from MSD curves fitting. More than 16 coacervates were analyzed per condition. Data are presented as mean ± SEM. Statistical significance between conditions is indicated by the asterisk (*p < 0.05, ***p < 0.001).

To investigate the motility behavior across different nanomotor surface coverages, we analyzed changes in MSD curves of coacervate droplets by extracting the anomalous diffusion exponent (α), by fitting the MSD to the equation (MSD = *K*Δ*t* ^α^) (Figure S19).^40^ Bare coacervates showed a linear MSD, indicating subdiffusive motion. This limited α (measured α was 0.76) likely arises from sedimentation effects due to gravity acting on relatively large coacervate droplets, which biases the motion away from ideal Brownian diffusion. It could also be due to the interactions between coacervates and the bottom slide, increasing drag and limiting diffusivity. A similar limited α of particles was also reported in previous work.^40^ For 30-Coas (nearly full nanomotor coverage), the measured α was 0.96, which indicates that upon laser irradiation of 30-Coas, a shift in motion dynamics from subdiffusive behavior toward more linear MSD is occurring, the observed diffusion enhancement in the 30-Coas case likely results from a combination of increased thermal agitation, and the fact that coverage is not perfectly uniform. Our 3D confocal imaging data confirm that even with 30-Coas, the nanomotor distribution is not absolutely complete, possibly allowing for minor local asymmetries (Figure 3j). However, this effect is not enough to generate a net displacement. In contrast, there was a clear shift from subdiffusive behavior toward enhanced Brownian motion (superdiffusion, α > 1) in the systems of 5-Coas and 15-Coas (measured α values were 1.02 and 1.08, respectively), consistent with active motility driven by an asymmetric distribution of nanomotor patches that produce autonomous motion via self-thermophoresis. The above motion analysis supports our self-thermophoretic mechanism that requires asymmetric heating.^41^

After confirming that we could modulate the motility of coacervates by tuning the distribution of the nanomotors on their surface, we aimed to investigate the impact of the coacervate size on the motion dynamics. We used two distinct methods to modify the size of coacervates, including sonication for small-sized coacervates and shaking for big-sized coacervates during formulation (Figure S20). As expected, the Brownian motion of smaller-sized 5-Coas was typically larger than that of the bigger-sized 5-Coas, showing higher MSD values (Figure 6a and Figure S21).^42^ With TP laser irradiation, the small-sized terpolymer-stabilized coacervates without nanomotors at the interface continued to exhibit Brownian motion due to the lack of active photothermal patches (Figure S22). However, both smaller-sized 5-Coas and bigger-sized 5-Coas displayed enhanced movement under laser irradiation due to asymmetric photothermal patches, with smaller-sized 5-Coas demonstrating higher MSD values, speed, and Deff (Figure 6a,b and Figure S23). In addition to size-dependent effects, the movement of light-driven micro/nano motors was also directly correlated with light intensity.^43–45^ Upon examining the motion behavior of bigger-sized 5-Coas under laser irradiation of increased intensity, a marked increase in the MSD profiles and corresponding speed were observed (Figure 6c,d and Figure S24). Furthermore, the trajectories of bigger-sized 5-Coas showed a directional trend as the laser intensity increased (Figure 6e,f). The motion dynamics of coacervates with nanomotors inserted into the terpolymer membrane was therefore consistent with the previously reported motion of light-driven motors.^46–48^

**Figure 6:**
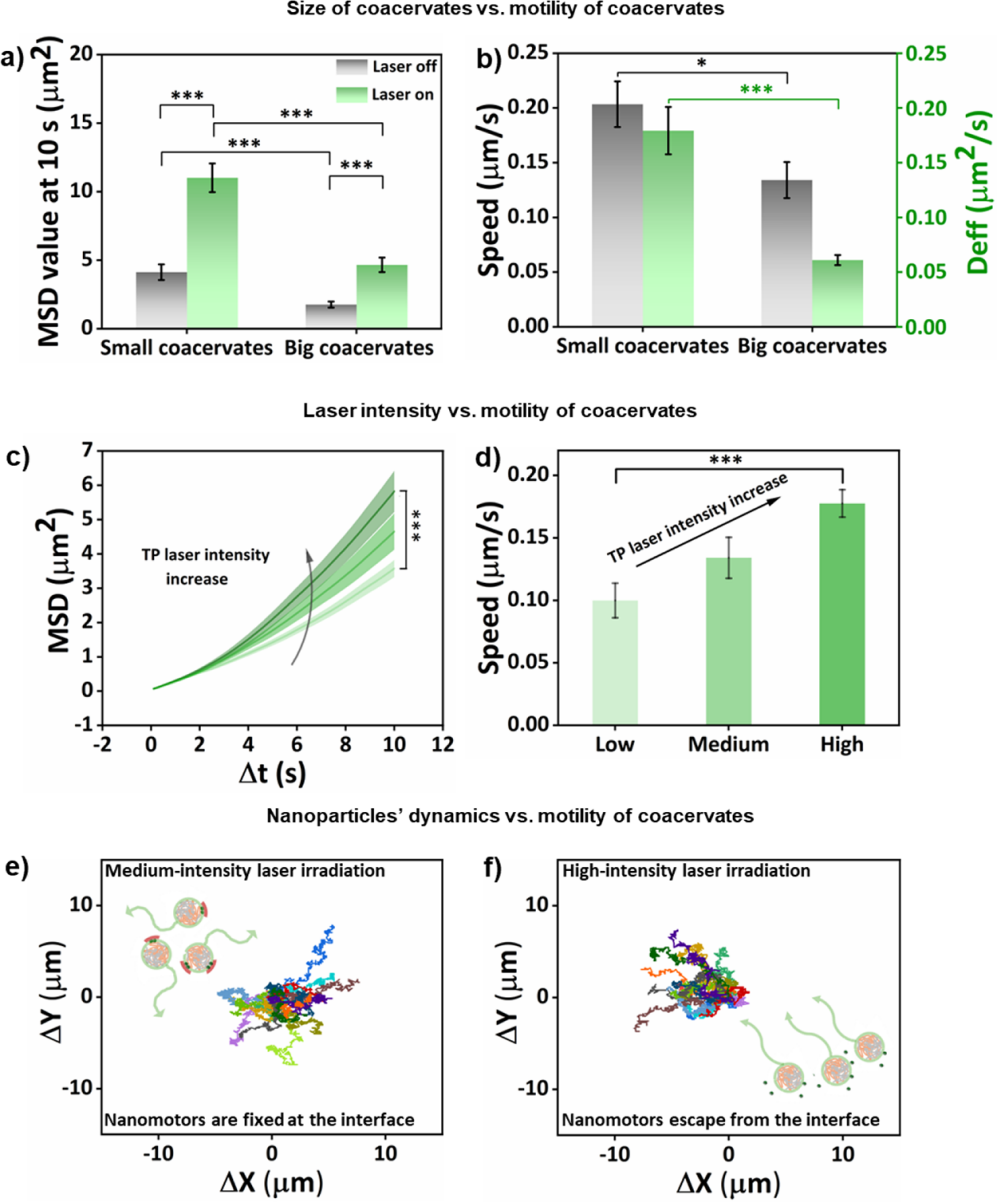
Investigation of motion dynamics depending on the coacervate size, laser intensity, and nanoparticles’ dynamics. (a) MSD values of smaller-sized and bigger-sized 5-Coas in a time frame of 10 s without and with TP laser irradiation. (b) Speed and Deff values of smaller-sized and bigger-sized 5-Coas with TP laser irradiation. MSD curves (c) and speed (d) of bigger-sized 5-Coas under TP laser irradiation at different laser intensity. Trajectories of bigger-sized 5-Coas under medium-intensity (e) and high-intensity (f) TP laser irradiation. More than 16 coacervates were analyzed per condition. The size of smaller-sized coacervates was about 1.9 μm. Data are presented as mean ± SEM. Statistical significance between conditions is indicated by the asterisk (*p < 0.05, ***p < 0.001).

We furthermore investigated the nanomotors’ position at the interface of coacervates, and we found that they were still firmly anchored after more than 2 min of medium-intensity laser irradiation. However, nanomotors tended to be released from the interface under high-intensity laser irradiation during the same irradiation time (Figure S25). The loss of nanomotors did not affect coacervate stability as the fluid terpolymer could still maintain the integrity of the coacervates. Although motility was still observed (Movie S4), reduced MSD values upon prolonged irradiation demonstrated a diminished motility due to the escape of nanomotors from the interface. The intensity of the laser upon prolonged irradiation thus affected motility, as only at the highest laser intensity studied the nanomotors reached sufficient kinetic energy to be released from the coacervate interface (Figure S26).

## Conclusions

In this study, we developed a coacervate-based artificial cell, which was stabilized by a hybrid membrane composed of terpolymer and gold nanoparticle-decorated stomatocytes. The stomatocytes were fixated at the interface in a stochastic, patchy fashion. By exploring the photothermal features of the gold nanoparticles and their asymmetric distribution, we could induce motile behavior in the coacervates. The movement of these coacervates was controlled by adjusting the nanomotor arrangement, coacervate size, and laser intensity. Furthermore, the presence of nanomotors significantly reduced the membrane’s permeability. This work presents an innovative strategy for stabilizing coacervates and offers a new approach for assembling micro- and nanomaterials to construct motile artificial cells, laying the foundation for significant advances in synthetic biology.

## Supplementary Material











## Notes

Syntheses of modified amyloses, terpolymer, PEG_22_PDLLA_95_ and PEG_44_PDLLA_95_; preparation of PEG-PDLLA stomatocytes, AuNPs-coated PEG-PDLLA stomatocytes, and Layer-by-Layer-modified PEG-PDLLA stomatocytes as well as terpolymer-stabilized coacervates without or with nanomotors at the interface; fluorescence recovery; quantification of dextran uptake; motility test; ^1^H-NMR and GPC data; SEM image; average size and zeta potential; CLSM images of hybrid coacervates; interfacial adsorption; 3D confocal z-stack image; stabilizing effect of different amounts of nanomotors; storage ability; size distribution of coacervates; analysis of trajectories, MSD values, speed, and effective diffusion coefficient of motile coacervates (PDF)
The motion of Coas and 5-Coas without and with medium-intensity (30%) TP laser irradiation (AVI)
A z-stack movie of a representative 5-Coas droplet was acquired to examine potential changes in the nanomotor patch configuration without medium-intensity (30%) TP laser irradiation and after completing a 2 min light-driven motility process (AVI)
The motion of 15-Coas and 30-Coas without and with medium-intensity (30%) TP laser irradiation (AVI)
The motion of 5-Coas without and with high-intensity (45%) TP laser irradiation (AVI)
