# Supplementary material for: Engineering Motile Coacervate Droplets via Nanomotor Stabilization
Source: J Am Chem Soc. 2025 Aug 20;147(35):31871–81. doi: 10.1021/jacs.5c09366 (PMC12412170; doi:10.1021/jacs.5c09366)
Supplement: Supplementary file 1 [file ja5c09366_si_001.pdf]

Supplementary information

Engineering motile coacervate droplets via nanomotor stabilization

Siwen Sun[†], Jianhong Wang[†], Yudong Li[†], Alexander B. Cook[†], Bingbing Sun[†], Sebastian Novosedlik[†], Lars J. M. M. Paffen[†], Sander G. A. M. Huisman[†], Lou M. V. Raeven[†], Alexander D. Fusi[†], Yunqi Guo[§], Loai K. E. A. Abdelmohsen[†], Shukun Li[†,], Tania Patiño Padial[†,*], Jan C. M. van Hest[†,*]*

[†]Bio-Organic Chemistry, Departments of Biomedical Engineering and Chemical Engineering & Chemistry, Institute for Complex Molecular Systems, Eindhoven University of Technology, 5600 MB Eindhoven, The Netherlands.

[§]State Key Laboratory for Modification of Chemical Fibers and Polymer Materials, Shanghai Engineering Research Center of Nano-Biomaterials and Regenerative Medicine, College of Biological Science and Medical Engineering, Donghua University, Shanghai, 201620, PR China

Contents

1. Materials

2. Instruments

3. Synthesis and methods

3.1 Synthesis of modified amyloses

3.2 Synthesis of terpolymer

3.3 Synthesis of $PEG_{22}PDLLA_{95}$ and $PEG_{44}PDLLA_{95}$

3.4 Preparation of PEG-PDLLA stomatocytes

3.5 Preparation of AuNPs coated stomatocytes

3.6 Preparation of Layer-by-Layer modified stomatocytes

3.7 Preparation of nanomotors-stabilized coacervates and nanomotors inserted into terpolymer-stabilized coacervates

3.8 Fluorescence recovery after photobleaching of nanomotors at coacervates' interface

3.9 Quantification of dextran uptake with confocal laser scanning microscopy

3.10 Motility test for coacervates

4. Supplementary figures

Figure S1. ^{1}H-NMR and GPC data of PEG-PDLLA copolymers.

Figure S2. SEM image of nanomotors.

Figure S3. Average size and zeta-potential of stomatocytes, nanomotors, stomatocytes loaded with Cy5.5, and nanomotors loaded with Cy5.5.

Figure S4. CLSM images of nanomotors inserted into terpolymer-stabilized coacervates.

Figure S5. CLSM images of nanomotors-stabilized coacervates and line intensity profiles.

Figure S6. CLSM images of nanomotors-stabilized coacervates. Nanomotors loaded with FITC and DiD.

Figure S7. Coacervates stabilized by LbL-modified stomatocytes and zeta-potential of modified stomatocytes.

Figure S8. The interfacial adsorption of bare PEG-PDLLA stomatocytes at the coacervate interface was examined by increasing the positive charge of the coacervates through varying the Q-Am to Cm-Am ratio.

Figure S9. 3D confocal z-stack image of nanomotors-stabilized amylose based coacervates (top view).

Figure S10. Bright field and confocal field tracking of the breakup process of intact coacervates droplets (nanomotors absorbed at the interface) and the subsequent release of nanomotors.

Figure S11. Stabilizing effect of different amount of nanomotors on coacervates.

Figure S12. 3D confocal z-stack images of nanomotors-stabilized amylose based coacervates.

Figure S13. Fluorescence intensity line profiles along the perimeter of 5-Coas, 15-Coas, and 30-Coas droplets.

Figure S14. Storage ability of nanomotors inserted into terpolymer-stabilized coacervates.

Figure S15. Permeability studies of a range of differently sized dextran using terpolymer-stabilized coacervates without and with nanomotors inserted into the terpolymer membrane.

Figure S16. Size distribution of 5-Coas, 15-Coas, and 30-Coas.

Figure S17. The trajectories of a representative 5-Coas: 30 s without laser irradiation and 2 min with medium-intensity TP laser irradiation.

Figure S18. MSD curves of 5-Coas, 15-Coas, and 30-Coas with and without medium-intensity TP laser irradiation.

Figure S19. MSD curves were fitted with MSD = $K\Delta t^{\alpha}$ to obtain the anomalous exponent α of Coas, 30-Coas, 15-Coas, and 5-Coas.

Figure S20. CLSM figures and size distribution of small-sized 5-Coas prepared by sonication for 4 minutes.

Figure S21. MSD curves of small-sized 5-Coas and big-sized 5-Coas without and with TP laser irradiation.

Figure S22. MSD curves of small-sized bare coacervates without and with TP laser.

Figure S23. The trajectories of small-sized 5-Coas and big-sized 5-Coas: 30 s without laser irradiation and 30 s with medium-intensity TP laser irradiation.

Figure S24. MSD curves of big-sized 5-Coas without and with TP laser irradiation at different laser intensities.

Figure S25. CLSM figures of 5-Coas without and with high-intensity laser irradiation and medium-intensity laser irradiation.

Figure S26. MSD curves of big-sized 5-Coas without and with medium-intensity and high-intensity TP laser irradiation. With laser irradiation, MSD curves at different time periods were analyzed.

5. References

Other supplementary materials for this manuscript include the following:

Movie S1. The motion of Coas and 5-Coas without and with medium-intensity (30%) TP laser irradiation.

Movie S2. A z-stack movie of a representative 5-Coas droplet was acquired to examine potential changes in the nanomotor patch configuration without medium-intensity (30%) TP laser irradiation and after completing a 2-minute light-driven motility process.

Movie S3. The motion of 15-Coas and 30-Coas without and with medium-intensity (30%) TP laser irradiation.

Movie S4. The motion of 5-Coas without and with high-intensity (45%) TP laser irradiation.

1. Materials

All materials were ordered from Sigma-Aldrich without further purification unless otherwise stated.

For PEG-PDLLA synthesis:

Poly(ethylene glycol) methyl ether 1K and 2K were purchased from Rapp Polymers and freeze-dried before use. D,L-lactide (DLL) was purchased from Acros Organics. 1,4- Dioxane and tetrahydrofuran (THF) were obtained from Biosolve Chimie. Dialysis Membrane MWCO (12,000 - 14,000 Da) was supplied from Spectra/Pro®.

For the preparation of coacervates:

Monomethoxy poly(ethylene glycol) 1 and 2 kDa were purchased from Rapp Polymere, trimethylene carbonate was purchased from TCI Europe.

For the preparation of modified amylose derivatives:

Amylose (12-16 kDa) was supplied by Carbosynth and 3-chloro-2- hydroxypropyltrimethyl ammonium chloride (65 wt% in water) was supplied by TCI Europe.

DBCO-Cy5.5 was purchased from Lumiprobe. DBCO-Cy3 and phosphate-buffered saline (PBS) were procured from Thermo Fisher Scientific. Poly(allylamine hydrochloride) (PAH) and poly(styrene sulfonate) (PSS) were purchased from Sigma Aldrich.

Ultrapure Milli-Q (Millipore) water (18.2 MΩ·cm) was used in this work.

2. Instruments

Nuclear Magnetic Resonance Spectroscopy (NMR):

Routine proton nuclear magnetic resonance (^1H NMR) measurements were performed on a Bruker AV 400 MHz Ultrashield^{TM} spectrometer, using $CDCl_3$ as the solvent and TMS as the internal standard.

Gel Permeation Chromatography (GPC):

The molecular weights (Mw, Mn) and dispersity index (Đ) of the block polymers were measured using a Prominence-I GPC system (Shimadzu) with a PL gel 5 μm mixed D column (Polymer Laboratories), equipped with a RID-20A differential refractive index detector. Polystyrene standards were used for calibration. THF was used as the eluent with a flow rate of 1 mL/min.

Dynamic Light scattering (DLS):

The hydrodynamic size, dispersity index (PDI), and zeta potential of the nanoparticles were determined by a Malvern instruments Zetasizer (model Nano ZSP) equipped with a 633 nm He-Ne laser and avalanche photodiode detector. Zetasizer software was further used to analyze the data.

Scanning Electron Microscopy (SEM):

Morphology of the nanoparticles was determined by a FEI Quanta 200 3D FEG.

Cryogenic Transmission Electronmicroscopy (cryo-TEM):

Cryo-TEM experiments were performed on the TU/e CryoTitan (Thermo Fisher Scientific) equipped with a field emission gun and autoloader and operated at 300 kV acceleration voltage in low-dose bright-field TEM mode. Samples for cryo-TEM were prepared by glow discharging the grids(Quantifoil Cu grid with R 2/2 holey carbon films, Quantifoil Micro Tools GmbH, part

of the SPT Life Sciences group) in a Cressington 208 carbon coater for 40 s. Then, 3 μL of samples (ca. 2.3 mg/mL) was pipetted on the grid and blotted in a Vitrobot MARK IV at room temperature and 100% humidity. The grid was blotted for 3 s (offset-3) and directly plunged and vitrified in liquid ethane. TEM images were acquired in zero loss energy filtering mode (Gatan GIF 2002, 20 eV energy slit) with a CCD camera (Gatan model 794).

Thermometer:

Temperature profiles of the coacervate solution (0.2 mL in a 1.5 mL Eppendorf tube) during irradiation were recorded with a thermometer (Chauvin Arnoux, C.A 1823).

Confocal laser scanning microscopy (CLSM):

Confocal laser scanning microscopy (Leica TCS SP8) was used for analysis of coacervates with fluorescent cargo. The system was equipped with a 488 nm laser (used for FITC), 552 nm laser (used for Nile red and Cy3), and 638 nm laser (used for DiD and Cy5.5) and a hybrid detector (HyD).

Two Photon-Confocal Laser Scanning Microscopy (TP-CLSM):

All motion videos were recorded using a CLSM (Leica TCS SP8X) equipped with 800 nm two-photon laser source (Chameleon Vision, Coherent, USA).

3. Synthesis and methods

3.1 Synthesis of modified amyloses

Both quaternized (Q-Am) and carboxymethylated (Cm-Am) amylose were prepared in accordance with previously published procedures[1-2].

Amylose was dissolved in aqueous NaOH solution. To prepare Q-Am, a solution of (3-chloro-2-hydroxypropyl)trimethylammonium chloride (60 wt% in water) was added dropwise to the stirring reaction mixture, which was then left to react overnight. For the preparation of Cm-Am, chloroacetic acid was added, and the mixture was stirred for 2 h. Both products were purified by precipitation into cold ethanol, followed by extensive dialysis against ultrapure water and then lyophilization.

3.2 Synthesis of terpolymer

Synthesis of poly(ethylene glycol)-b-poly(ε-caprolactone)-g-poly(trimethylene carbonate)-b-poly(glutamic acid) (PEG_{44}- b-PCL_{50}-g-$PTMC_{50}$-b-PGA_{8}) terpolymer

This block terpolymer was synthesized as described previously[2-3].

ε-Caprolactone and trimethylene carbonate were polymerized via ring-opening polymerization, initiated by poly(ethylene glycol) monomethyl ether and catalyzed by methanesulfonic acid. The polymer's terminal alcohol was then modified via Steglich esterification with Boc-L-Phe-OH, yielding a primary amine after TFA deprotection. The poly(L-glutamic acid) block was subsequently introduced through the ring-opening polymerization of N-carboxyanhydride γ-benzyl L-glutamate, followed by hydrogenolysis.

3.3 Synthesis of $PEG_{22}PDLLA_{95}$ and $PEG_{44}PDLLA_{95}$

The reaction was carried out using previously published procedures[4].

Briefly, poly(ethylene) glycol (PEG) was weighed into a flame-dried 50 mL round bottom flask. D,L-lactide (DLLA, 95 eq.) was added and all reagents were subsequently dried by co-evaporation with toluene. Thereafter, dry dichloromethane (DCM) was added under argon. Subsequently, 1,8-diazo-bicyclo[5.4.0]undec-7-ene was added to initiate the ring opening polymerization. The reaction was left stirring under Ar atmosphere at RT for 2 h. Disappearance of monomer peaks was monitored using ^{1}H-NMR spectroscopy. The reaction mixture was diluted with DCM and extracted with $KHSO_4$, water and brine. The organic layer was collected and dried with Na_2SO_4, filtered and concentrated in vacuo. The resulting oil was precipitated in ice-cold diethyl ether and lyophilized from dioxane to yield a white powder.

3.4 Preparation of PEG-PDLLA stomatocytes

The stomatocytes were prepared following previously published procedures[4].

In a 20 mL vial, PEG_{22}-$PDLLA_{95}$ and PEG_{44}-$PDLLA_{95}$ block copolymers (1:1 w/w%, 10 mg) were weighed in and dissolved in 1 mL THF/dioxane (volume ratio=1:4). The vial was equipped with a stirring bar and sealed with a rubber septum. A needle was inserted to release pressure. The solution was stirred at 900 rpm for 30 min, followed by addition of 1 mL ultrapure water with a syringe pump at a rate of 1 mL/h. The solution was then dialyzed overnight against 75 mM NaCl at 4 °C with an ultrapure water change after 1 h to yield stomatocytes. For fluorescent stomatocytes: Cy5.5-DBCO (0.01 mg), FITC (0.1 mg), or DiD (0.01 mg) was added to the organic solvent mixture separately before dissolving the copolymers.

3.5 Preparation of AuNPs coated stomatocytes

The AuNPs coated stomatocytes (nanomotors) were prepared in accordance with previously published procedures[5].

In a 20 mL vial, poly(acrylic acid) (PAA, 0.8 mg) and $HAuCl_4 \cdot 4H_2O$ (1.2 μL, 1 mg mL^{-1}) were added to 1 mL PEG-PDLLA stomatocytes solution. The mixture was stirred at a rate of 500 rpm for 10 min at RT. Then, a solution of $NaBH_4$ (1 mL, 5 mM) was added dropwise and the reaction mixture was stirred for another 10 min at RT. The solution was centrifuged down, supernatant was removed and MilliQ water was added. This solution was washed three times. Finally, the precipitate was redispersed in 1 mL Milli-Q. Samples were stored in the 4 °C fridge for further use.

3.6 Preparation of Layer-by-Layer modified stomatocytes

Poly(allylamine hydrochloride) (PAH) and poly(styrene sulfonate) (PSS) solutions were prepared at a concentration of 2 mg/mL, 6 mg/mL, and 10 mg/mL using 0.1 M NaCl. First, 0.8 mL PAH solution was added to the PEG-PDLLA stomatocytes templates (0.8 mL, 3.33 mg/mL), and the mixture was shaken for 20 min at room temperature. Then, 0.1 M NaCl was used to wash the stomatocytes and remove excess PAH by centrifugation (12,000 rpm, 7 minutes). The mixture was shaken for another 20 min at room temperature after addition of 0.8 mL PSS solution, followed by centrifugation at 12,000 rpm for 5 min to remove all the supernatant after which the samples was washed with Milli-Q water three times. Finally, the precipitate was redispersed in Milli-Q. Samples were stored in the 4 °C fridge for further use.

3.7 Preparation of nanomotors-stabilized coacervates and nanomotors inserted into terpolymer-stabilized coacervates

For nanomotors-stabilized coacervates, Q-Am and Cm-Am were dissolved separately in phosphate-buffered saline at a concentration of 1 mg/mL. Coacervation was induced by mixing the solutions of Q-Am and Cm-Am in a ratio of 2:1. For a typical experiment to create

nanomotor-stabilized coacervates, 66 μL of Q-Am solution was added to a 1.5 mL Eppendorf tube containing 33 μL of Cm-Am solution and 50 μL 3.33mg/mL nanomotor solution while stirring in an Eppendorf tube shaker at 1500 rpm for 4 min. For preparation of nanomotors inserted into terpolymer-stabilized coacervates, coacervation was induced by mixing the solutions of 66 μL of Q-Am , 33 μL of Cm-Am, and different volumes of nanomotor solution in an Eppendorf tube shaker at 1500 rpm for 4 min. After 4 min of stirring, 3 μL of terpolymer stock solution (50 mg/mL in PEG350) was slowly added to stabilize the coacervate droplets.

3.8 Fluorescence recovery after photobleaching of nanomotors at coacervates' interface

To check the mobility of nanomotors, fluorescence recovery after photobleaching (FRAP) (Leica TCS SP8 confocal microscope) was performed on the nanomotor patch. A selected circular area was bleached, and the fluorescence intensity in the bleached area was monitored over time. The coacervate droplets with a diameter of 18-21 μm were selected. 10 images were acquired prior to the bleaching. Subsequently, the ROI (d = 3 μm for nanomotors inserted into terpolymer-stabilized coacervates) was bleached. The recovery was monitored with a 5 s interval and 70 images were collected for each experiments. The intensities of the bleached ROI, reference area, and background were extracted from the images with FIJI. Data was analyzed with Origin, following a literature procedure[3]. Fluorescence intensity at time t, I(t) was background subtracted and corrected for unintentional photobleaching, which was calculated as

$$\mathbf{I(t)} = \frac{\mathbf{ROI(t) - B_g}}{\mathbf{Re\,f(t) - B_g}}$$

where ROI(t) is the average intensity of the bleached area at time t, Ref(t) is the average intensity of an unbleached fluorescent area (same size as bleached area) and B_g is the average

intensity of a background area (same size as bleached area). I(t) was further normalized such that pre-bleach intensity was set to 1. The results were obtained by measuring 3 different droplets. Data are presented as mean ± standard deviation.

3.9 Quantification of dextran uptake with confocal laser scanning microscopy

Hybrid coacervates were prepared as described under coacervate preparation. The coacervate suspension (30 μL) was transferred to an Ibidi 18 well μ-slide and was diluted with 1xPBS buffer (30 μL). Then the respective FITC-dextran (either 4, 40 or 500 kDa; 1 μL, 1 mg mL^{-1}) was added to the well and measured with CLSM after 10 min and 24 hours of incubation time. The degree of permeability was then determined with confocal microscopy by quantifying the encapsulation efficiency of the fluorescent dextran. The average fluorescent emission values of FITC-dextran measured inside a number of coacervate protocells was measured by FIJI, and compared to the same area outside the coacervate protocells. For all experiments n=30 coacervates were measured across multiple imaging positions in the same sample.

3.10 Motility test for coacervates

Experimental chamber

The same experimental chamber was constructed as reported previously[6]. The chamber was assembled using two glass microscopy slides separated by two strips of autoclave tape. To create the chamber, autoclave tape was affixed to both ends of a larger glass slide. The sample was then placed in the center, and the setup was sealed with a smaller glass slide on top.

Optical recording

The videos of coacervate motion were recorded using a CLSM (Leica TCS SP8X) equipped with two-photon laser source (Chameleon Vision, Coherent, USA). A 63×oil immersion

objective was used for this recording. For the video recording, the zoom factor was 4.5. Coacervates were loaded with FITC to visualize the coacervates with fluorescence. The coacervate suspension was diluted 6 times and immediately added to the chamber. The intensity of the TP laser was set to 15% (low), 30% (medium), and 45% (high). The laser irradiation area was selected to be approximately 40 μm*40 μm rectangular area (ROI) to eliminate possible interference from solution. The recording started shortly after. Videos were recorded at a rate of 10 FPS, with a recording period of 5–35 s without laser irradiation and 10–40s with laser irradiation. As a control groups, 15 μL nanomotors was mixed with 100 μL terpolymer stabilized coacervates to eliminate the influence of free nanomotors in solution on the movement of coacervates.

Data analysis of motion

A custom Python script was employed to track the movement of coacervates and calculate their mean square displacement (MSD). The MSD quantifies how far a particle deviates from its initial position over time, providing insight into its dynamics. This metric is widely used to study the behavior of self-propelled particles and is determined in a 2D projection using the following formula:

$$\boldsymbol{MSD}(\boldsymbol{t}) = \left\langle \left(\vec{\boldsymbol{\chi}}(\boldsymbol{t}) - \vec{\boldsymbol{x}}(\boldsymbol{0})\right)^{2} \right\rangle$$

where $\vec{\boldsymbol{\chi}}(\boldsymbol{0})$ is the initial position of the coacervate, and $\vec{\boldsymbol{\chi}}(\boldsymbol{t})$ is the position of the coacervate when time is t. The obtained MSD was plotted against the time interval Δt. To extract the velocity of the particles in the self-propulsive regime, a quadratic fitting to the equation $\mathrm{MSD}(\Delta t) = 4D_{\mathrm{T}}\Delta t + V^2\Delta t^2$ was performed. For each experimental condition, more than 16 coacervates were analyzed to ensure robust data collection and accurate motion assessment.

4. Supplementary figures

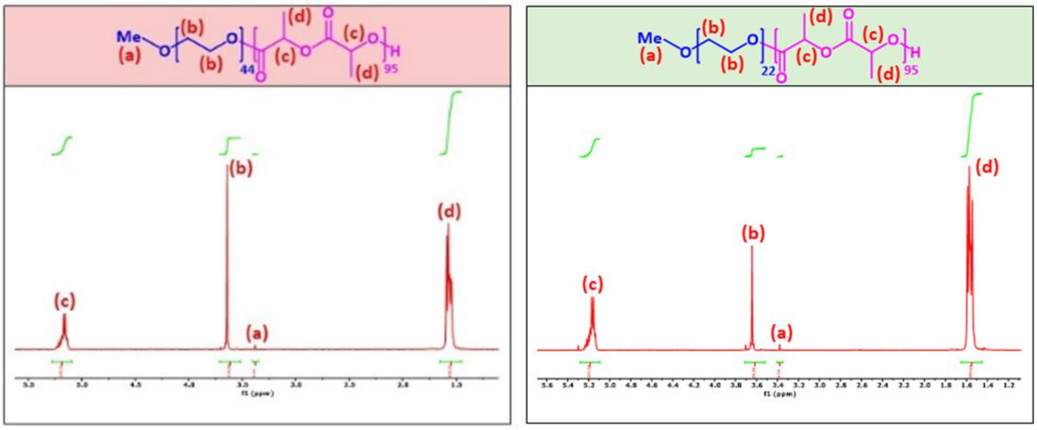

Polymer	PEG$_{44}$-PDLLA$_{95}$
Composition by ^{1}H-NMR	PEG$_{44}$-PDLLA$_{90}$
Mn from GPC (kg/mol)	12.9
Mw from GPC (kg/mol)	13.6
Đ from GPC	1.05

Polymer	PEG$_{22}$-PDLLA$_{95}$
Composition by ^{1}H-NMR	PEG$_{22}$-PDLLA$_{97}$
Mn from GPC (kg/mol)	11.7
Mw from GPC (kg/mol)	12.4
Đ from GPC	1.06

Figure S1. ^{1}H-NMR and GPC data of PEG-PDLLA copolymers.

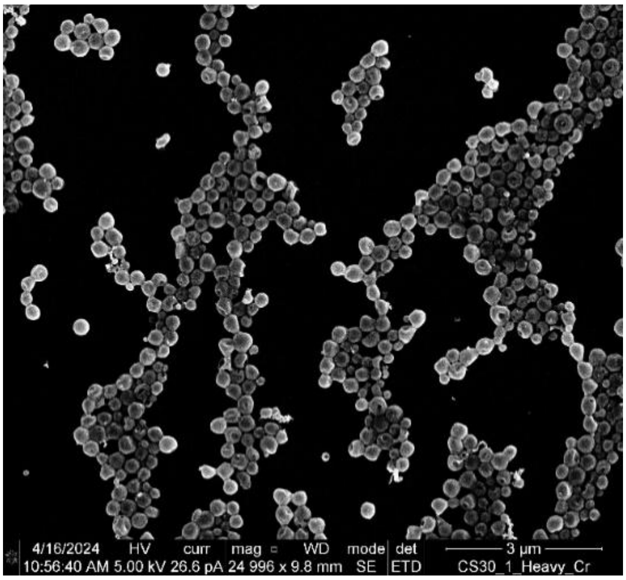

Figure S2. SEM image of nanomotors.

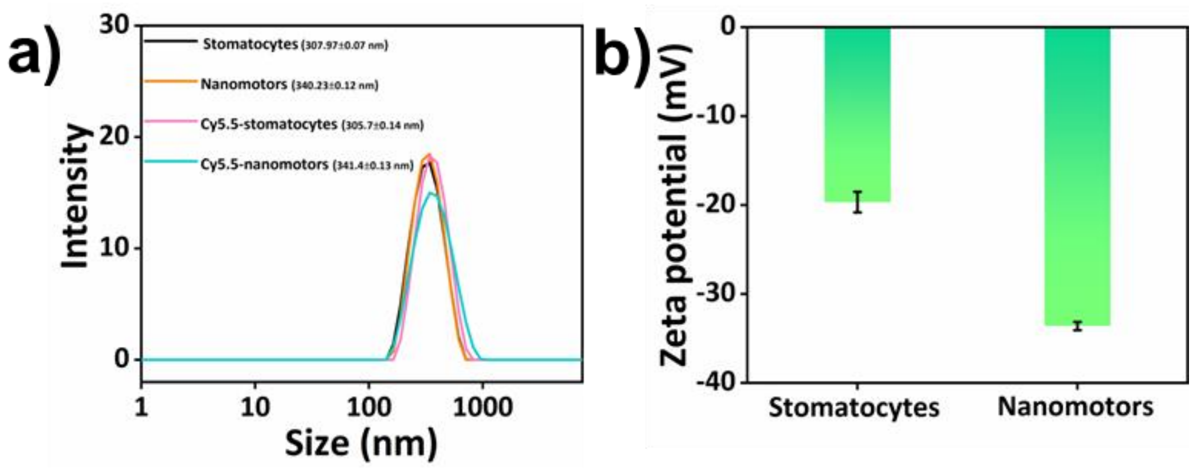

Figure S3. (a) Average size of stomatocytes, nanomotors, stomatocytes loaded with Cy5.5, and nanomotors loaded with Cy5.5. (b) Zeta potential of stomatocytes and nanomotors.

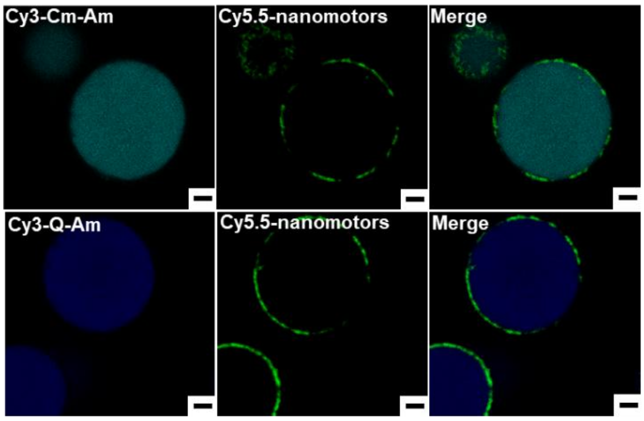

Figure S4. CLSM images of nanomotors inserted into terpolymer-stabilized coacervates. Composition is 33 µL 1 mg/mL Cm-Am solution, 20 µL 3.33 mg/mL nanomotor solution, and 66 µL 1 mg/mL Q-Am solution, which are mixed by shaking for 4 min, after which terpolymer is added. Cyan: Cy3 conjugated Cm-Am, Blue: Cy3 conjugated Q-Am. The terpolymer here was not dye-labeled. Images were adjusted for brightness and contrast solely for visualization of each component. No quantitative analysis was performed on these processed images. Scale bars represent 2 µm.

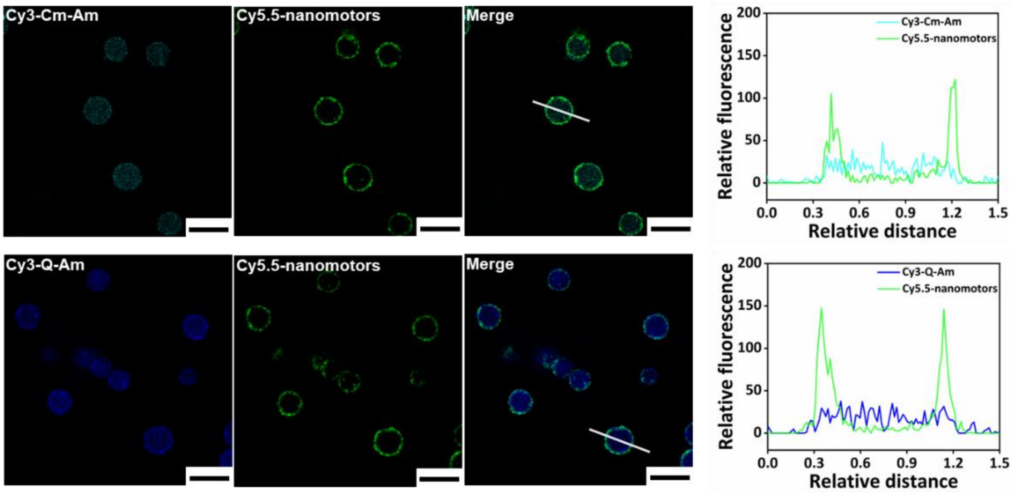

Figure S5. CLSM images of nanomotors-stabilized coacervates and related line intensity profiles. Samples were prepared by mixing 66 μL 1 mg/mL Q-Am solution, 33 μL 1 mg/mL Cm-Am solution, and 50 μL 3.33 mg/mL nanomotor solution by shaking 4 min (1500 rpm). Green: nanomotors loaded with Cy5.5, Cyan: Cy3 conjugated Cm-Am, Blue: Cy3 conjugated Q-Am. Images were adjusted for brightness and contrast solely for visualization of each component. The data analysis of line intensity profiles was performed using raw, unprocessed images. Scale bars represent 10 μm.

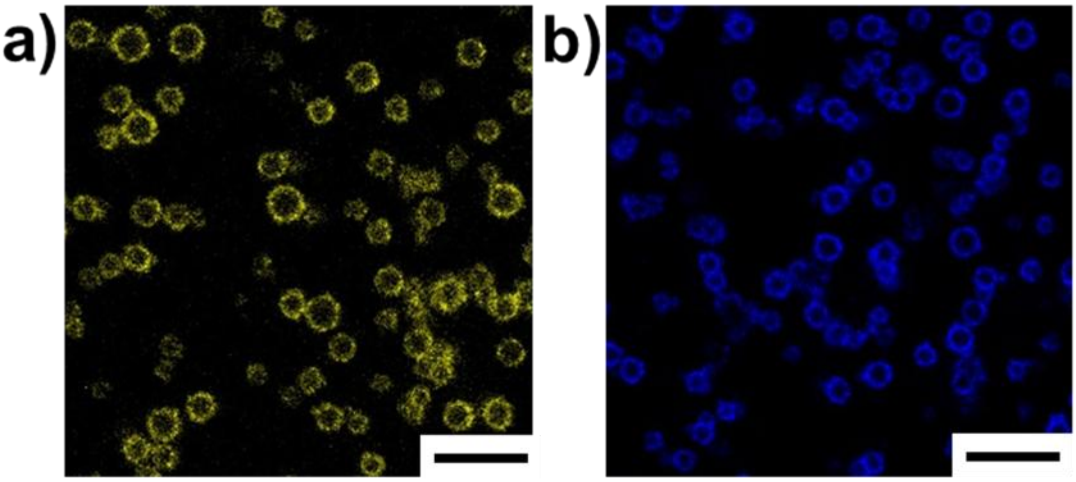

Figure S6. CLSM images of nanomotors-stabilized coacervates. Nanomotors loaded with FITC (a) and DiD (b). Samples were prepared by mixing 66 μL 1 mg/mL Q-Am solution, 33 μL 1 mg/mL Cm-Am solution, and 50 μL 3.33 mg/mL nanomotor solution by shaking 4 min (1500 rpm). Scale bars represent 10 μm. Images were adjusted for brightness solely for visualization.

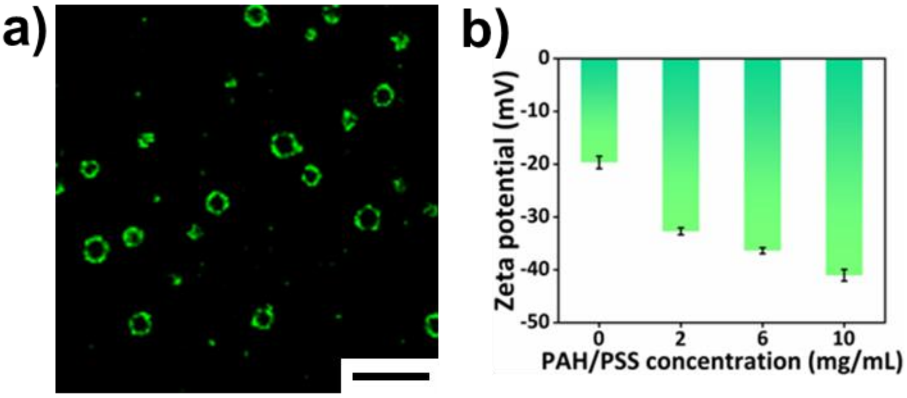

Figure S7. (a) Coacervates stabilized by LbL-modified stomatocytes. The concentration of the polymers for the LbL process (PAH/PSS) was 6 mg/mL. Samples were prepared by mixing 66 µL 1 mg/mL Q-Am solution, 33 µL 1 mg/mL Cm-Am solution, and 50 µL 3.33 mg/mL modified stomatocytes solution by shaking 4 min (1500 rpm). Green: nanomotors loaded with Cy5.5. Scale bar represents 10 µm. (b) Zeta-potential of LbL-modified stomatocytes at different polymer concentrations.

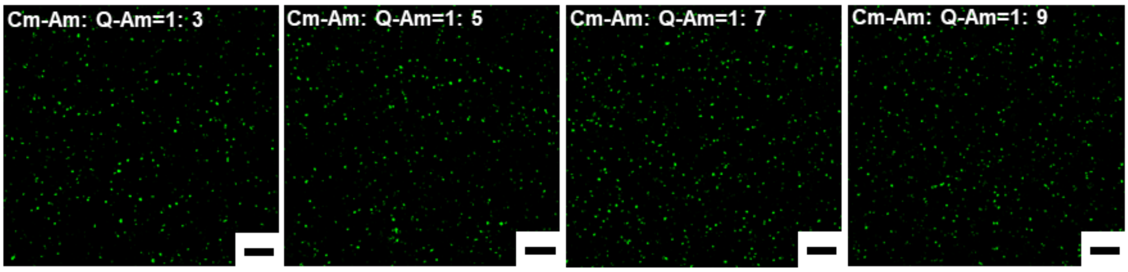

Figure S8. The interfacial adsorption of bare PEG-PDLLA stomatocytes at the coacervate interface was examined by increasing the positive charge of the coacervates through varying volume ratio of Q-Am to Cm-Am. Samples were prepared by mixing 1 mg/mL Q-Am solutions, Cm-Am solutions, and 50 µL 3.33 mg/mL PEG-PDLLA stomatocytes solutions, followed by shaking at 1500 rpm for 4 minutes. The volumes of Q-Am solution used were 75 µL, 83 µL, 87.5 µL, and 90 µL, while the corresponding volumes of Cm-Am solution were 25 µL, 17 µL, 12.5 µL, and 10 µL, respectively. Green: nanomotors loaded with Cy5.5. Scale bar represents 10 µm.

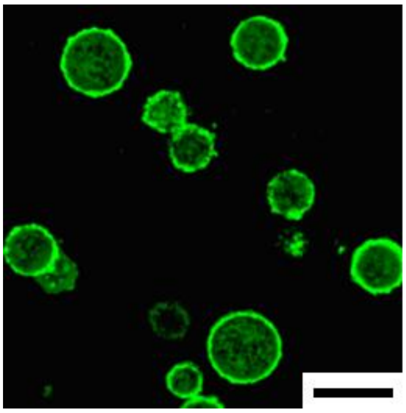

Figure S9. 3D confocal z-stack image of nanomotors-stabilized amylose based coacervates (top view). Green: nanomotors loaded with Cy5.5. Scale bar represents 10 μm. Composition is 33 μL 1mg/mL Cm-Am solution, 50 μL 3.33 mg/mL nanomotor solution, and 66 μL 1mg/mL Q-Am solution, which are mixed by shaking for 4 minutes.

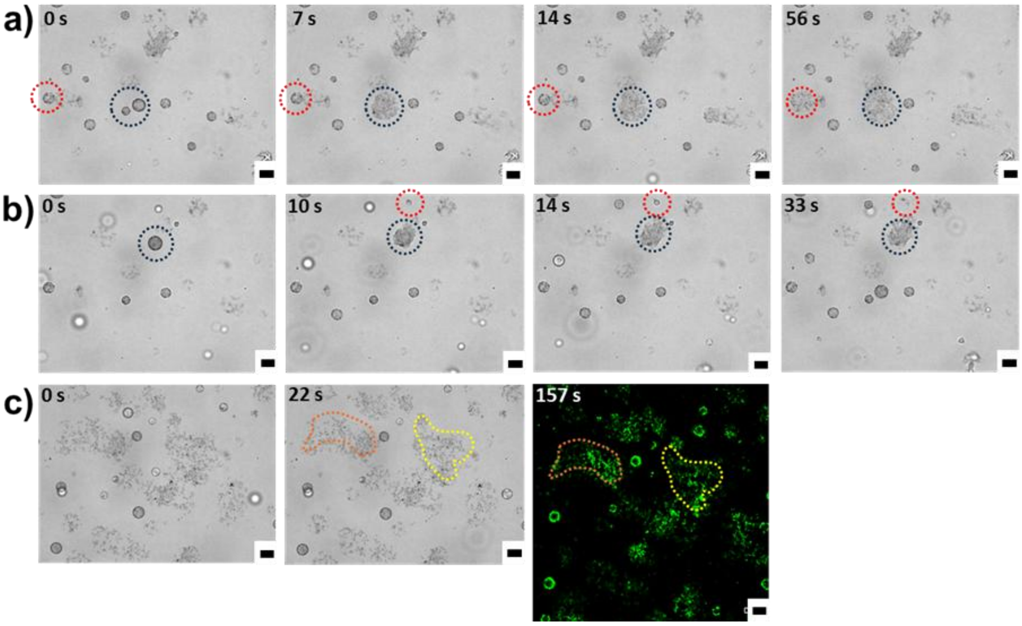

Figure S10. Bright field and confocal field tracking of the breakup process of intact coacervate droplets (nanomotors absorbed at the interface) and the subsequent release of nanomotors. The black and red dotted circles more clearly show the breakup process of a complete droplet. The orange and yellow dashed areas represent the nanomotors released after a droplet collapse. Composition is 33 μL 1 mg/mL Cm-Am solution, 20 μL 3.33 mg/mL nanomotor solution, and 66 μL 1 mg/mL Q-Am solution which are mixed by shaking for 4 minutes. Green: nanomotors with encapsulated Cy5.5. Scale bars represent 10 μm.

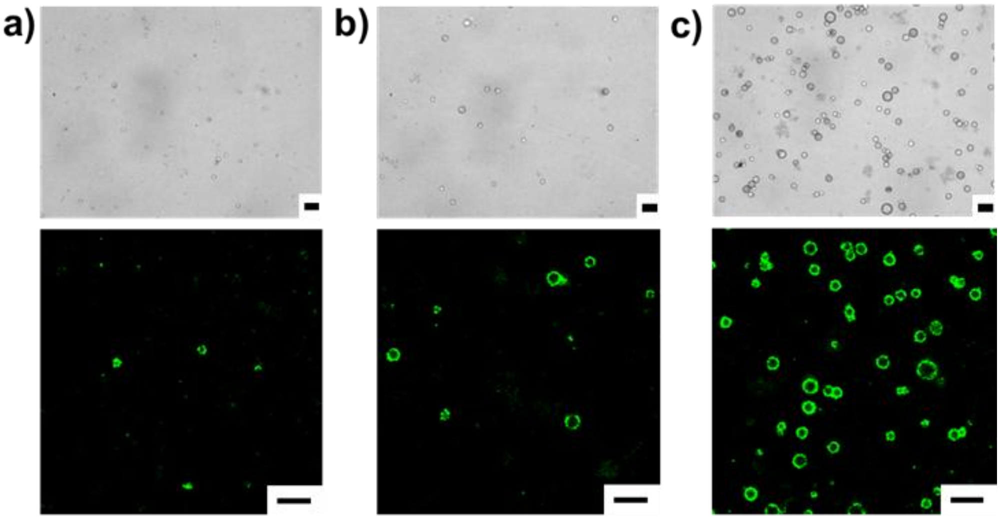

Figure S11. Nanomotors-stabilized coacervates. Composition is 33 µL 1 mg/mL Cm-Am solution, 3.33 mg/mL nanomotor solution, and 66 µL 1 mg/mL Q-Am solution, which are mixed by shaking for 4 minutes, the volume of introduced nanomotor solution is (a) 5, (b) 15, and (c) 30 µL respectively. Scale bars represent 10 µm.

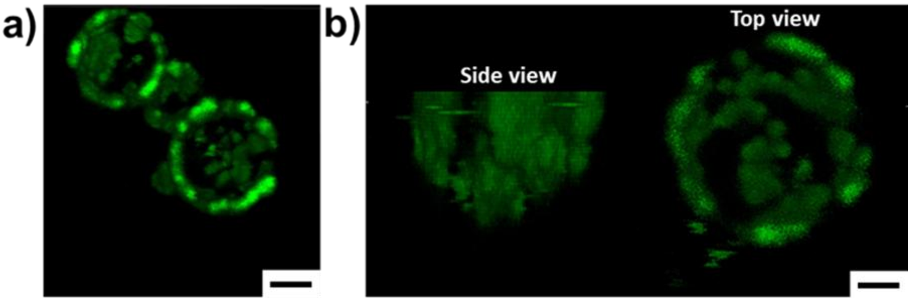

Figure S12. 3D confocal z-stack images of nanomotors-stabilized amylose-based coacervates. Composition is 33 µL 1mg/mL Cm-Am solution, 15 µL 3.33 mg/mL nanomotor solution, and 66 µL 1mg/mL Q-Am solution, which are mixed by shaking for 4 minutes. Green: nanomotors loaded with Cy5.5. Scale bars represent 2 µm (a) and 1 µm (b).

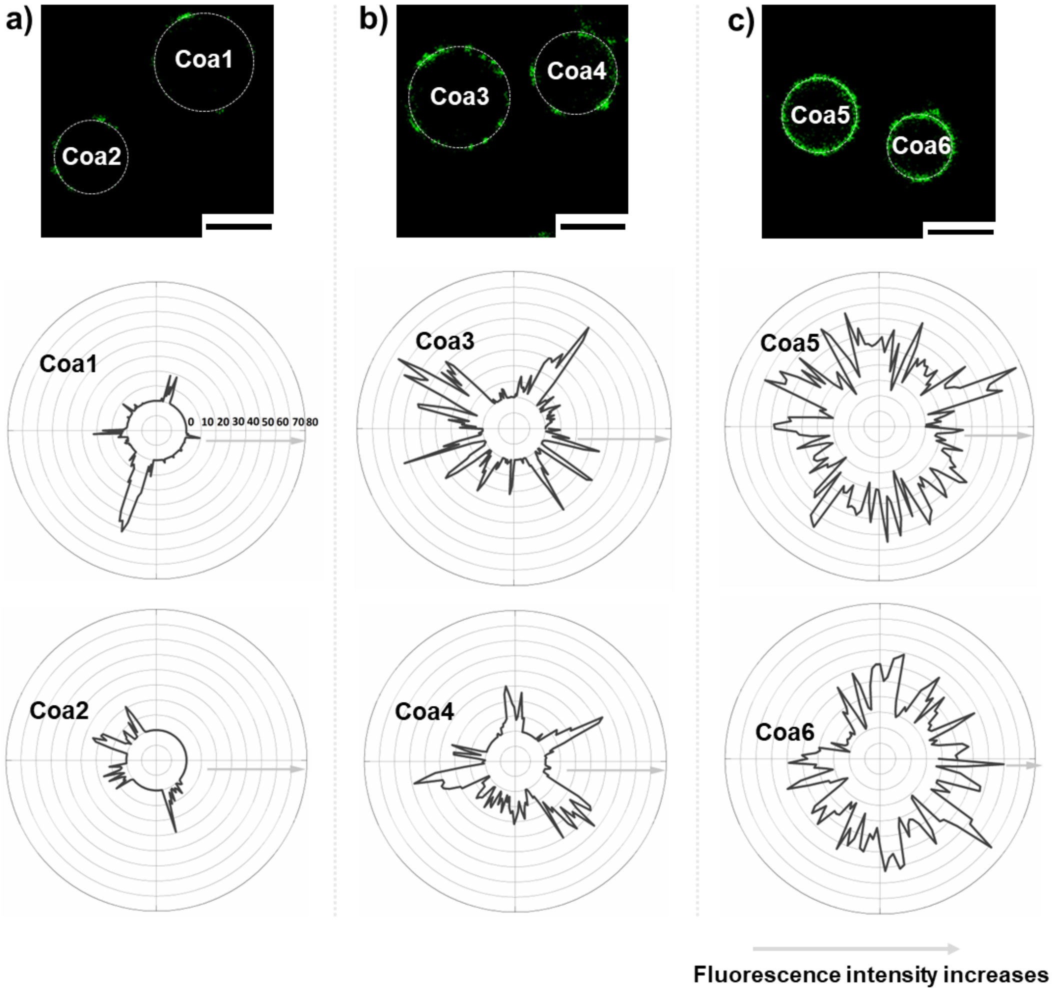

Figure S13. Fluorescence intensity line profiles along the perimeter of 5-Coas, 15-Coas, and 30-Coas droplets. The gray arrow indicates a gradual increase in fluorescence intensity. These images are used solely to qualitatively indicate the presence and approximate distribution of nanomotors on the droplet surface at the selected z-plane. Due to the relatively large size of the nanomotors (over 340 nm), the fluorescence intensity does not directly reflect their quantity. Some nanomotors may lie slightly above or below the focal plane, and not all may be in perfect focus, which can affect the apparent fluorescence signal. Line profile analysis was performed using raw, unprocessed images.

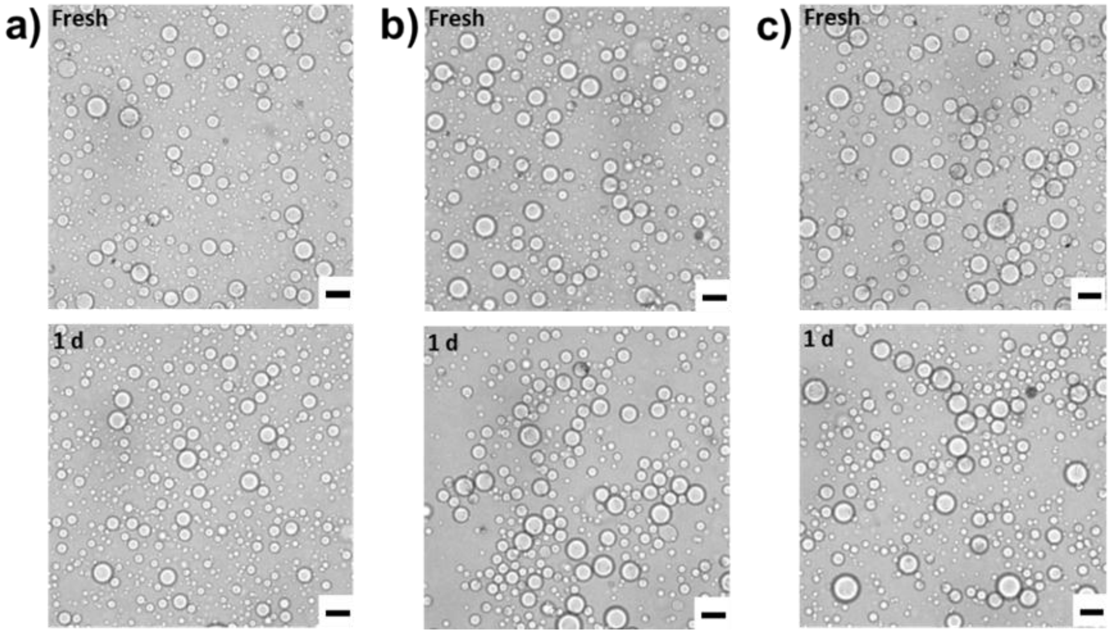

Figure S14. Storage ability of nanomotors inserted into terpolymer-stabilized coacervates. Composition is 33 μL 1 mg/mL Cm-Am solution, 3.33 mg/mL nanomotor solution, and 66 μL 1 mg/mL Q-Am solution, which are mixed by shaking for 4 min, after which terpolymer is added. The volume of introduced nanomotor solution is (a) 5, (b) 15, and (c) 30 μL, respectively. Scale bars represent 10 μm.

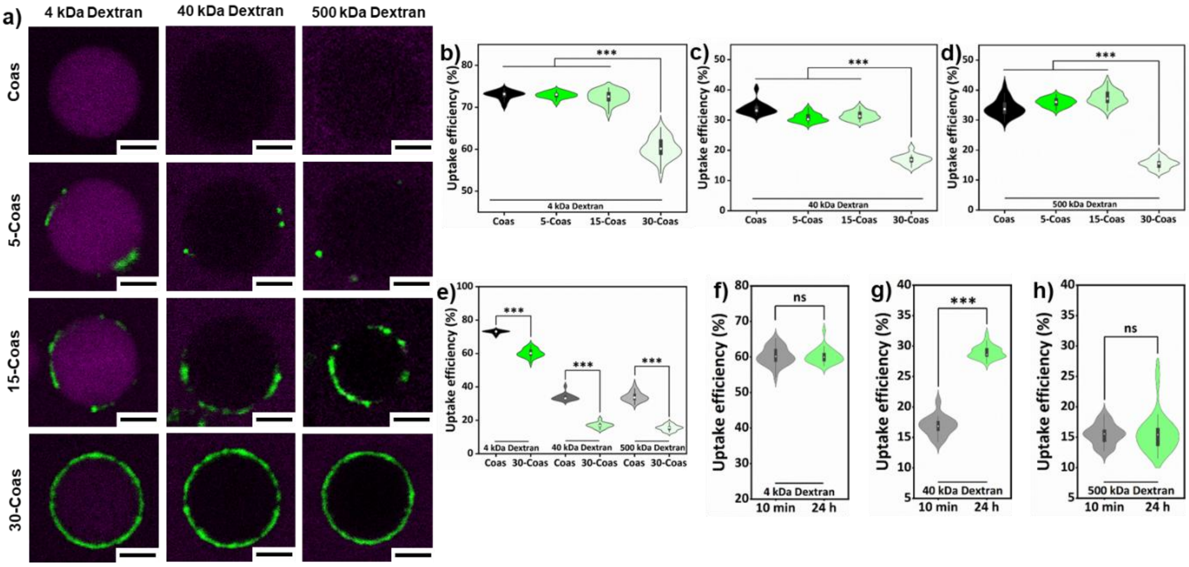

Figure S15. Permeability studies of a range of differently sized dextran using terpolymer-stabilized coacervates without and with nanomotors inserted into the terpolymer membrane. (a) CLSM images of terpolymer-stabilized coacervates and coacervates with nanomotors inserted into the terpolymer membrane exposed to 4 kDa FITC-dextran, 40 kDa FITC-dextran, and 500 kDa FITC-dextran after 10 min exposure. Quantification of sequestration efficiency of 4 kDa FITC-dextran (b), 40 kDa FITC-dextran (c), and 500 kDa FITC-dextran (d) after 10 min exposure. (e) Uptake efficiency of 4 kDa FITC-dextran, 40 kDa FITC-dextran, and 500 kDa FITC-dextran in terpolymer-stabilized coacervates and coacervates with a membrane highly loaded with nanomotors (30-Coas) after 10 min exposure. Uptake efficiency of (f) 4 kDa FITC-dextran, (g) 40 kDa FITC-dextran, and (h) 500 kDa FITC-dextran in 30-Coas followed over time. Terpolymer-stabilized coacervates with 0 μL 3.33 mg/mL nanomotor at the interface (Coas), 5 μL 3.33 mg/mL nanomotor at the interface (5-Coas), 15 μL 3.33 mg/mL nanomotor at the interface (15-Coas), and 30 μL 3.33 mg/mL nanomotor at the interface (30-Coas). For all experiments n=30 coacervates were measured. Scale bars represent 3 μm. FITC-dextran (magenta) and Cy5.5-nanomotors (green). Statistical significance between conditions is indicated by the asterisk (***$p < 0.001$). For all violin plots the black box represents interquartile range (IQR), the black line represents 1.5x IQR and the white circle represents the median. Data of permeability analysis were performed using raw, unprocessed images.

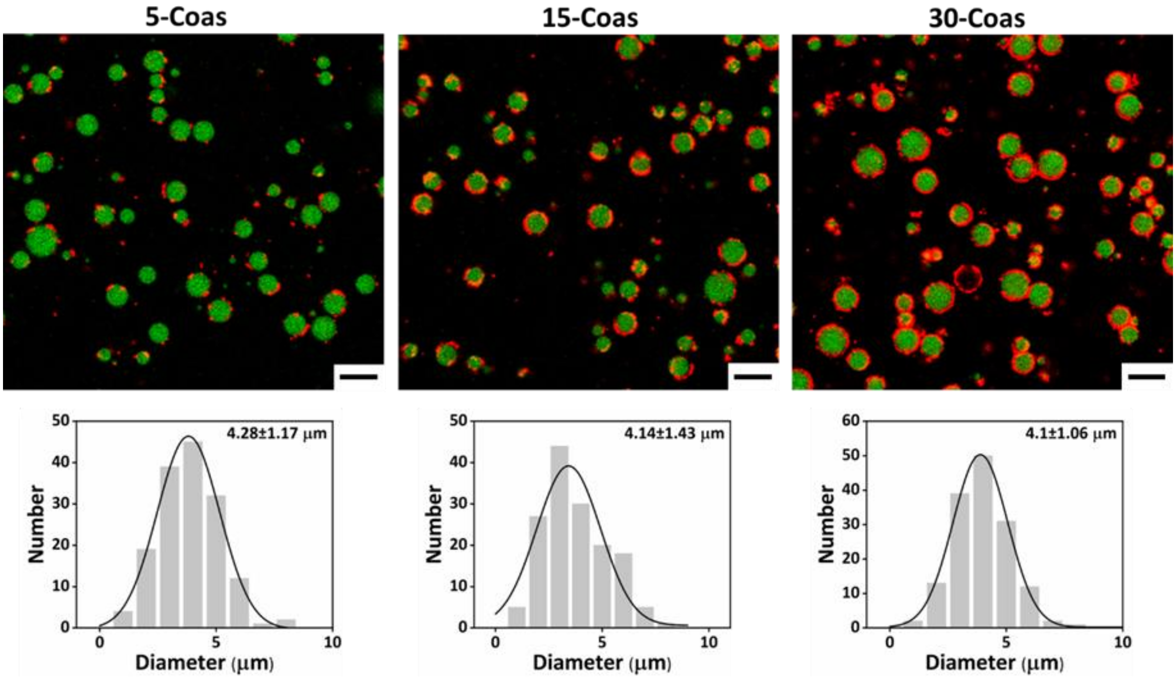

Figure S16. Size distribution of 5-Coas, 15-Coas, and 30-Coas. Composition is 33 μL 1 mg/mL Cm-Am solution, 3.33 mg/mL nanomotor solution, and 66 μL 1 mg/mL Q-Am solution, which are mixed by shaking for 2 min, after which terpolymer is added. The volume of introduced nanomotor solution is 5, 15, and 30 μL respectively. Scale bars represent 10 μm.

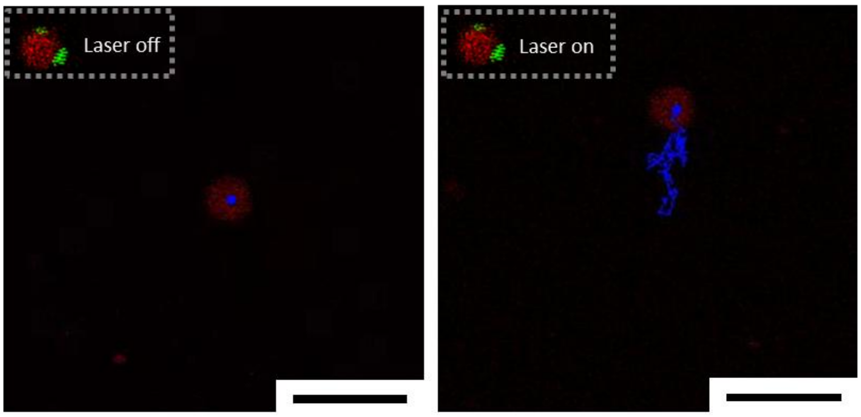

Figure S17. The trajectories of a representative 5-Coas: 30 s without laser irradiation and 2 min with medium-intensity TP laser irradiation. Scale bars represent 10 μm.

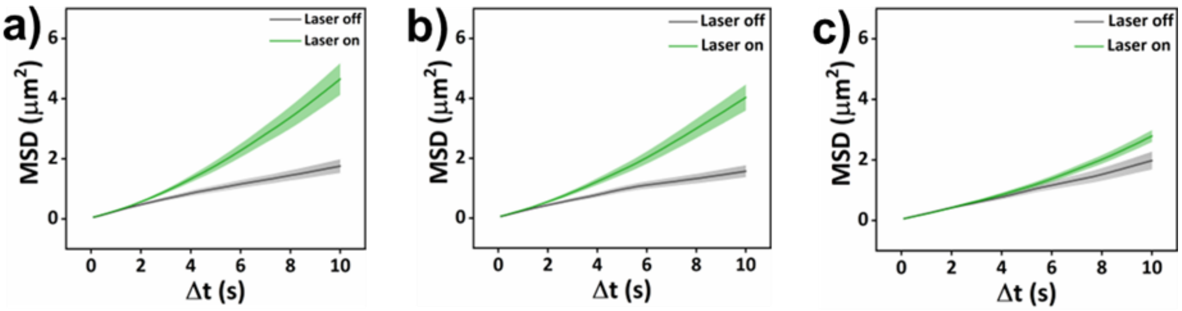

Figure S18. MSD curves of 5-Coas (a), 15-Coas (b), and 30-Coas (c) with and without medium-intensity (30%) TP laser irradiation.

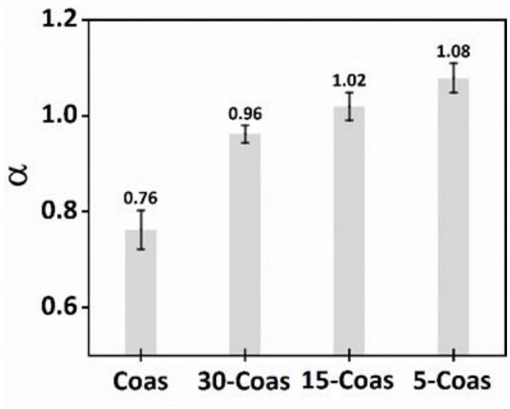

Figure S19. MSD curves were fitted with MSD = $K\Delta t^{\alpha}$ to obtain the anomalous exponent α of Coas, 30-Coas, 15-Coas, and 5-Coas.

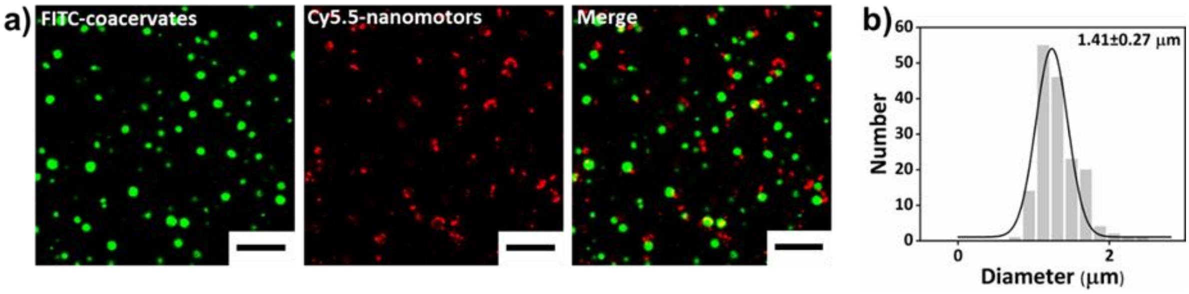

Figure S20. CLSM figures and size distribution of small-sized 5-Coas prepared by sonication for 4 minutes. Scale bars represent 10 μm.

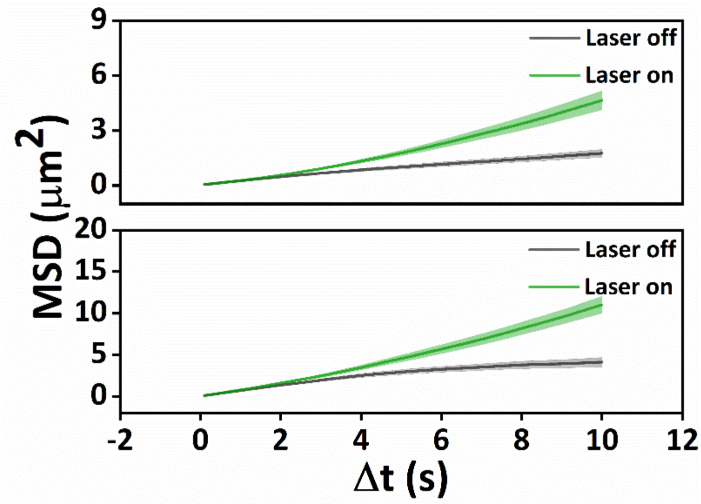

Figure S21. MSD curves of small-sized 5-Coas (bottom) and big-sized 5-Coas (top) without and with TP laser irradiation (30% laser intensity for big-sized 5-Coas and 25% intensity for small-sized 5-Coas).

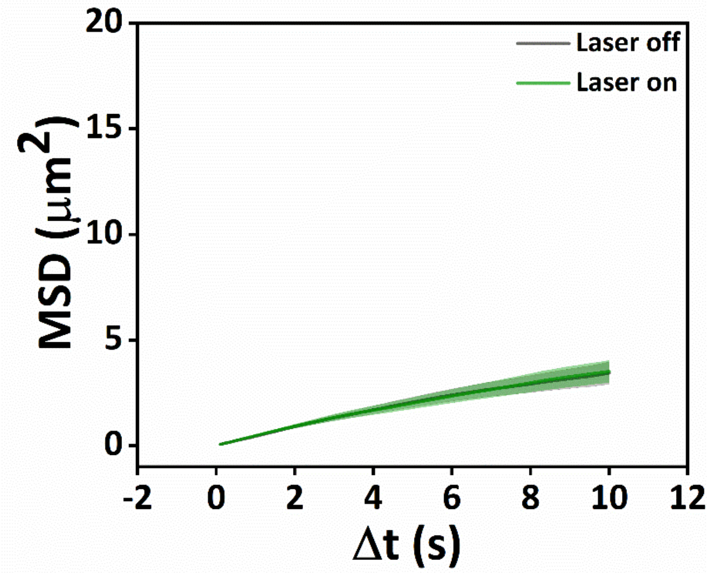

Figure S22. MSD curves of small-sized bare coacervates without and with TP laser (25% intensity) irradiation.

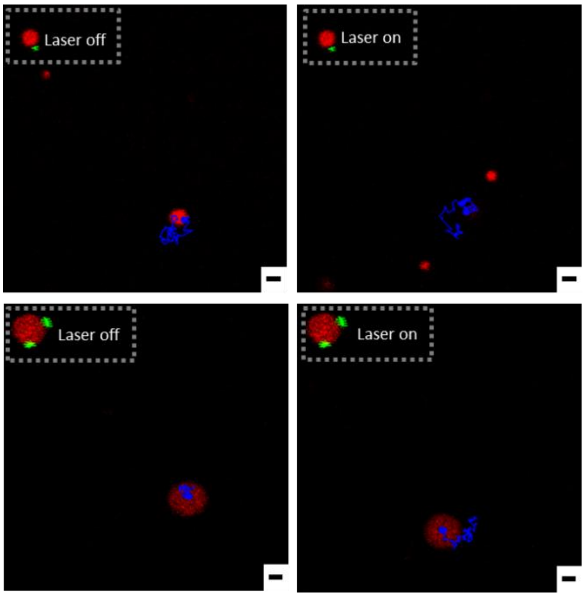

Figure S23. The trajectories of small-sized 5-Coas and big-sized 5-Coas: 30 s without laser irradiation and 30 s with medium-intensity TP laser irradiation. Scale bars represent 1 μm.

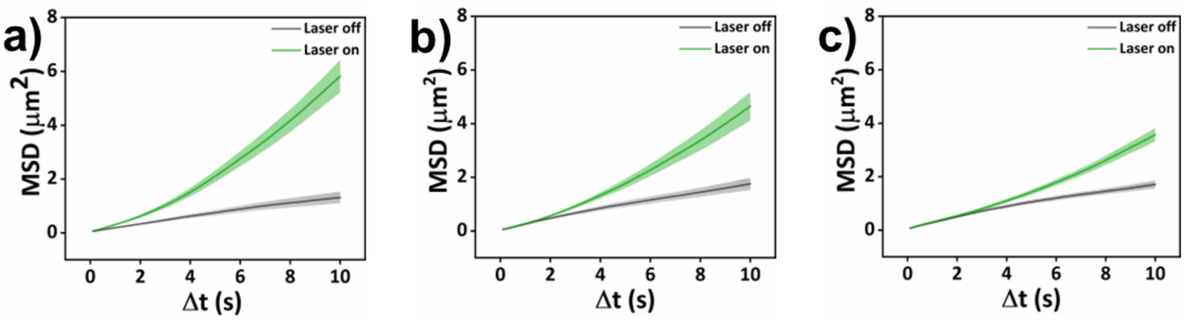

Figure S24. MSD curves of big-sized 5-Coas without and with TP laser irradiation at different laser intensities. (a) high laser intensity (45%), (b) medium laser intensity (30%), and (c) low laser intensity (15%).

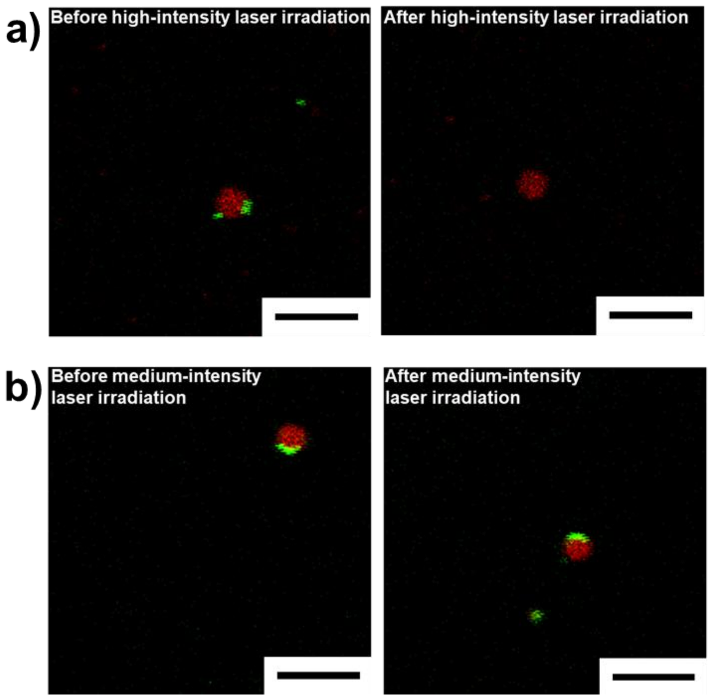

Figure S25. CLSM figures of 5-Coas without and with high-intensity (45%) laser irradiation (a) and medium-intensity (30%) laser irradiation (b). The laser irradiation time was the same, about 135 minutes. Scale bars represent 10 μm.

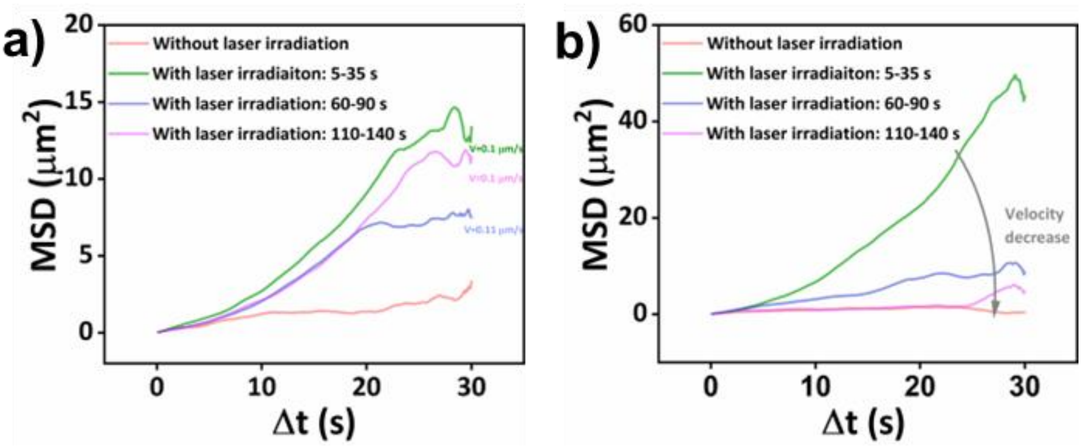

Figure S26. MSD curves of big-sized 5-Coas without and with medium-intensity (a) and high-intensity (b) TP laser irradiation. With laser irradiation, MSD curves at different time periods (5-35 s, 60-90 s, and 110-140 s) were analyzed. The corresponding coacervates' motion were shown in movie S1 (a) and S4 (b).

5. References

1. Mason, A. F.; Altenburg, W. J.; Song, S.; van Stevendaal, M.; van Hest, J. C. M., Terpolymer-Stabilized Complex Coacervates: A Robust and Versatile Synthetic Cell Platform. *Methods Enzymol.* **2021,** *646*, 51-82.

2. Mason, A. F.; Buddingh', B. C.; Williams, D. S.; van Hest, J. C. M., Hierarchical Self-Assembly of a Copolymer-Stabilized Coacervate Protocell. *J. Am. Chem. Soc.* **2017,** *139* (48), 17309-17312.

3. Novosedlik, S.; Cook, A. B.; Voermans, T. J. F. M.; Janssen, H. M.; van Hest, J. C. M., Control over Membrane Fluidity and Biophysical Properties of Synthetic Terpolymer Stabilized Complex Coacervates. *Polym. Chem.* **2024,** *15* (45), 4650-4661.

4. Pijpers, I. A. B.; Cao, S.; Llopis-Lorente, A.; Zhu, J.; Song, S.; Joosten, R. R. M.; Meng, F.; Friedrich, H.; Williams, D. S.; Sánchez, S.; van Hest, J. C. M.; Abdelmohsen, L. K. E. A., Hybrid Biodegradable Nanomotors through Compartmentalized Synthesis. *Nano Lett.* **2020,** *20* (6), 4472-4480.

5. Wang, J.; Wu, H.; Zhu, X.; Zwolsman, R.; Hofstraat, S. R. J.; Li, Y.; Luo, Y.; Joosten, R. R. M.; Friedrich, H.; Cao, S.; Abdelmohsen, L. K. E. A.; Shao, J.; van Hest, J. C. M., Ultrafast Light-Activated Polymeric Nanomotors. *Nat. Commun.* **2024,** *15* (1), 4878.

6. Song, S.; Mason, A. F.; Post, R. A. J.; De Corato, M.; Mestre, R.; Yewdall, N. A.; Cao, S.; van der Hofstad, R. W.; Sanchez, S.; Abdelmohsen, L. K. E. A.; van Hest, J. C. M., Engineering Transient Dynamics of Artificial Cells by Stochastic Distribution of Enzymes. *Nat. Commun.*

2021, *12* (1), 6897.
